# Sit—Writing

**Published:** 1883

**Authors:** 


**SIT up, must**: oppression of chest during cough, so great that patient must sit up in bed (also to expectorate), when she experiences great pain, with constrictive sensation under sternum: PHOS.

— — wheezing, panting on waking then violent cough which forces him to sit up and bend forward: Kali b.[7]

**SITTING**, cough when: Alum., Euphr., Ferr., Guai., Kali c., Mag. c., Mag. m., Natr. c., Phos., Ph. ac., Puls., Rhus,[7] Sabad., Sep., Zinc.—12.

— **bent**, excites cough: Spig., Stann.

— — forward, dry cough from tickling behind upper half of sternum, at 11 A. M., when: *Rhus.*

— **dry** cough, when: Agar., Phos.

— after **dinner**, dry cough, when: Agar.

— **erect**, cough when: Kali c., Natr. m.,[2] Spong.—12.

— **long** in same position, excites cough: Coc. c.,[2] Ph. ac.[12]

— **still**, cough while, afternoon: Coca.

— — agg. cough: Rhus.[7]

— — cough ceases only when: Verat.[6]

— **up, amel.** cough: Arg. n., Cinnab., Mag. s.,[6] HYOS., Natr. c., Phos., PULS., Rhus,[1] *Sang.*[1]—12.

— — amel. cough; coughs incessantly lying: Cinnab.

— — — constant cough lying at night, better on sitting up: *Hyos.*, *Puls.*

— — — dry cough: Arg. n., Cinnab., *Sang.*

— — — — — at night: *Puls.*

— — — exhausting cough: Natr. c.

— — — hollow cough: Natr. c.

**SITTING up**, amel. loose cough, about midnight: Phos.

— — — paroxysmal cough: Cinnab.

— — — rough cough: Natr. c.

— — — short cough: Arg. n., Cinnab.

**SKIN** troubles with cough: Bell., Con., Lach., Lact., Puls.—6.

— burning, biting in region of scurf (of ulcer), early in morning with a dry cough: Puls.

— scarlet spots and redness on face, neck, abdomen, etc., with quick pulse, asthma and violent cough: Bell.[6]

— œdematous swelling of body, dullness of head with asthmatic troubles and short cough: Lact.[6]

**SLEEP**, cough **after**: Lach.,[16] Puls.

— — severe and obstinate hoarseness, worse evening, with tickling in larynx and cough, especially after sleeping: Arum ital.

— — See on Waking.

— cough **before**: Merc.[12]

— **choking** cough, as if would suffocate, as soon as one falls into a sound sleep: LACH.[14]

— during **coryza**, dry cough in sleep: Sepia.

— **disturbed** by dry cough: Alum.,[6] Sang.[12]

— — cough at night prevents sleep and causes exhaustion: PULS.

— — cough which disturbs sleep, and is succeeded by a dry heat: Alum.[6]

— — incessant coughing prevents sleep: SEPIA.[1]

— — sleep is disturbed by frequent coughing and aching in the feet: *Sepia.*[6]

— — unable to sleep, at night, from excessive tormenting cough: *Rhus.*

**SLEEP**, cough **during**: *Acon.*, Arn., *Bell.*, Calc., Carbo an., *Cham.*, Hippo.,[1] Hyos., *Lach.*, Kreos., Merc., Samb., Sep., Stram., Verb. —12.

— **during**, barking cough: Nitr. ac.

— — dry cough: *Cham.*, Coff., Mag. s.

— — — — before midnight: *Nitr. ac.*, *Rhus.*

— — loud cough: Lyc.[10]

— — on lying down at night and in sleep, cough is absent: Kali b.[6]

— — cough at night: Calc.

— — cough at noon: Euphr.

— — severe cough, especially after lying down and sleeping: Apis.[7]

— — violent cough: Cyc., Sulph.

— **after going to**, evening suffocative cough: Carbo an., *Lach.*

— **before going** to, dry cough long time, evening in bed, before falling asleep, and worse than during day: SULPH.

— **on going to**, cough: Guarc., Sep., Sulph.

— — — —, dry cough evening: *Hepar.*

— — — — evening, agg. tickling cough: Lyc.

— — — — cough on going to sleep, with heat in the head and face, and cold hands: *Sulph.*

— — — — morning and evening, agg. exhausting cough: Lyc.

— **prevented.** See Disturbed.

— **restless**, from moderate cough, afternoon and night, with bloody expectoration: Mez.[6]

— on **starting** in, coughs: Apis.

— starting in, cough with: Cina, Hepar.—12.

**SLEEPLESSNESS** with cough: Benz. ac.,[16] Kreos., Nitrum.—12.

**SLEEPY** immediately after coughing: *Ant. t.*,[10] Ign.[19]

**SLEEPY**, violent convulsive cough (whooping cough), caused by tickling in pharynx, agg. at night and after eating; after the attacks yawning and sleepiness: Anac.[10]

**SMARTING** and burning causes cough: Zing.

**SMOKE** of all kinds excites cough: Euphr.,[2] Ment. pi.[1]

**SMOKING tobacco** excites cough: *Acon.*, All. s.,[1] Brom., Bry., Carbo an., Cham.,[16] Clem.,[5] Cocc., Coc. c.,[1] Coloc.,[16] Dros.,[16] Euphr.,[1] Ferr., Hell., Hepar, Ign., Iod., Lach., Mag. c., Ment. pi.,[1] Nux v., Osm.,[1] Petro., Spig.,[7] Spong., Staph., Sul. ac., Thuja.[1] —2.

— **amel.** convulsive cough: Tarent.

— — dry cough, at night: Tarent.

— — exhausting cough: Tarent.

— after **dinner**, cough on: Coc. c.

— **dry** cough from: All. s., Atro., Coc. c., Hell.[10]—1.

— — — evening: Thuja.

— **hacking** cough: Clem., Hell.

— — — evening: Coloc.

— **hard** cough, while: All. s.

— **interrupted** cough, evening: Thuja.

— **paroxysmal** cough, while: All. s.

— **periodic** cough, smoking or speaking: Atro.

— **short** cough: Coca.

— — — evening: Thuja.

— **tickling** cough: Atro.

— — dry cough: Coca.

**SMOTHERED** cough: Meli.

— feeling, with hacking cough at night: Asaf.

**SNEEZING after** coughing: *Agar.*,[1] Bad.,[2] Bell.,[16] *Hepar.*[6]

— — — cough appears in isolated attacks, is violent and ends with repeated sneezing: *Agar.*

— **excites** cough: Senega.[12]

**SNEEZING** with cough: Ant. t.,[11] Aspar.,[16] *Bell.*, Bry., Nitr. ac.,[11] Osm., Sep.[11]—12.

— — catarrh in throat that provokes cough: Petro.[7]

— — constant sneezing; sharp pains in centre of temples; tickling cough; light mushy stools: Iris v.[7]

— — wakes at night from sneezing, with tickling in throat causing cough: Amm. m.[7]

**SONOROUS** cough: Amm. cau., STRAM.

**SNORING** with cough: Ant. t., Arg., Bell., Caust., Ipec., Natr. c., Natr. m., Nux v., Puls., Sep.—12.

**SOCIETY** of strangers excites cough: Ambra, *Ars.*, Bar.—2.

**SORE** cough, evening: Calc. s.

— — morning: Cod.

**SORENESS** when coughing: Spig.

— — — See under Abdomen, Chest, Throat, etc.

**SOUNDING**, deep cough: Aloe,[1] Mang., Stann.—10.

**SOUNDLESS** cough: Dros.[12]

**SOUR food** excites coughing: Ant. c., Brom., Lach., Natr. m., Nux v., Sep., Sulph.—2.

**SPASMODIC** cough: Acon., Agar.,[7] All. c.,[2] *Ambr.*, Bad.,[2] Bar., *Bell.*, Brom., BRY., Cact., *Calc.*,[2] CARBO V., Cast., Chel., *China*, CINA, Con., Coral., CUPR., Dig., DROS., *Ferr.*, Ferr. acet., *Hepar*, HYOS., *Ign.*, *Iod.*, IPEC., Kali b., *Kali c.*, *Kreos.*, Lach., *Lact.*, Laur., Led., Lyc., *Mag. c.*, *Mag. m.*, *Merc.*, Mezer., Mosch., *Natr. m.*, Nitr. ac., NUX V., Osm., Phos., Ph. ac., *Plumb.*, PULS., Rhus, Samb., Sep., Sil., Squil., Staph., Stram., *Sulph.*, *Verat.*, Zinc.—5 and 11.

— — compare with Convulsive and Paroxysmal Coughs.

**SPASMODIC** cough, **afternoon** agg.: All. c., Bad., *Bell.*,[12] Bry.,[12] Mur. ac., Zinc.—2.

— — **amel.** by eating, morning: Ferr.[10]

— — **breath**, violent, spasmodic cough, commencing with gasping for breath, and continuing with repeated crowing inspirations, till face becomes black or purple and patient exhausted; agg. at night and after a meal: *Coral.*[2]

— — — violent, hollow, shattering spasmodic cough, excited by tickling in larynx, with suffocative arrest of breathing: Led.[2]

— — **chest**, **burst**, ungovernable spasmodic cough, which seems as if it would burst the thorax, excited by a peculiar tickling in pharynx, which again, is induced by a sensation of smothering in throat: Lact. v.[2]

— — — **constriction**, spasmodic cough, excited by a feeling like constriction of chest and trachea, as if from vapor of sulphur; worse from midday to midnight: Mosch.[2]

— — — **constriction**, periodically recurring attacks of painless, barking, spasmodic cough, in a shrill screeching tone, excited by constriction of chest and larynx; worse in morning: Stram.[2]

— — — **creeping** in, violent, short paroxysms of spasmodic cough, excited by mucus in trachea and by creeping in chest; worse after midnight and in morning: Squil.[2]

— — — **itching - scratching**, shattering, spasmodic whooping cough, often in paroxysms of two coughs each, excited by an itch-

**SPASMODIC**—(*Continued*).
ing scratching with a feeling of dryness and, as it were, of vapor of sulphur in chest and trachea; worse evening till midnight; expectoration in morning and daytime: Puls.[2]

— — — **chest**, **pain**, spasmodic cough, hollow and deep, with rawness, hoarseness, and pain through chest; with suffocative feeling when inhaling; cannot exhale; vomits all food some hours after eating; bloated face; convulsions; worse at night and lying: Meph.[10]

— — — **scratching**, hollow or whistling, spasmodic cough, excited roughness, scratching and tickling in chest and throat; worse morning and evening: Kreos.[2]

— — — **tickling itching**, hollow, hacking, spasmodic, tickling cough, excited particularly by a tickling itching in chest; worse evening and night; expectoration morning and by day: Phos.[2]

— — — — powerful, spasmodic, nocturnal paroxysms of whooping cough, excited by tickling and itching in chest and throat, or as if by dry spot in larynx; worse at night; difficult expectoration in day: Con.[2]

— — — **tickling**, spasmodic, shattering cough, excited by tickling creeping in larynx, in trachea, and in chest; worse evening until midnight; no expectoration in evening: Rhus.[2]

— — **day** and night: Sulph.[12]

— — **evening** agg.: All. c., Bad., Bell., Bry., Calc., Ign., Ipec., Lach., Laur., Merc., Natr. m., *Phos.*, Ph. ac., Sil.—2.

**SPASMODIC** cough **evening** until midnight, agg.: Bar., Carbo v., Ferr., Led., Mez., Nitr. ac., *Puls.*, Rhus, Sepia, Stann., Zinc.—2.

— — — until after midnight, agg.: Mag. c., Mag. m.—2.

— — **expectorating**, grayish phlegm in little lumps, morning: Natr. ars.

— — **forenoon**, agg.: Sabad., Sepia.—2.

— — **larynx**, **constriction** of, periodically recurring attacks of painless, barking spasmodic cough, in a shrill, screeching tone, excited by constriction of larynx and chest; worse in morning: Stram.[2]

— — — **constriction** of, spasmodic cough at night, occurring in paroxysms every quarter of an hour, each paroxysm consisting of but few coughs, with a rough, hollow, barking tone, excited by tickling in throat, as if from down or, as it were, by constriction of larynx; worse evening and night: Bell.[2]

— — — **down** in, spasmodic tickle cough, excited, as it were, by down in larynx, in suprasternal fossa and in chest as far as epigastrium; worse morning and evening: Ph. ac.[2]

— — — **irritation** in, spasmodic, violent whooping cough, excited by irritation from larynx to chest; worse evening till midnight; morning expectoration: Mez.[2]

— — — — spasmodic cough, always in two paroxysms which occur in rapid succession, excited by irritation in larynx and in upper chest; worse at night, or also in evening: day expectoration: Merc.[2]

**SPASMODIC** cough, **larynx, irritation** spasmodic, hollow whooping cough in short hard coughs and infrequent paroxysms, excited by feeling as if sulphur vapor were inhaled, or by a creeping irritation in larynx and throat; worse evening till midnight; morning expectoration: Carbo v.[2]

— — — **tickling** in, violent, hollow, shattering, spasmodic cough, excited by tickling in larynx, with suffocative arrest of breathing; worse evening until midnight; expectoration after midnight and in morning: Led.[2]

— — **tickling** creeping, spasmodic shattering cough, excited by tickling creeping in larynx, trachea, and chest; worse evening until midnight: Rhus.[2]

— — — — shattering, barking, spasmodic cough, excited by tickling in larynx and in epigastrium; worse evening till midnight, rarely only in day; expectoration morning and day: Nitr. ac.[2]

— — — — spasmodic cough, harsh, hoarse, dry ringing, excited by constant tickling in larynx (which is temporarily relieved by eating apples); the cough produces a raw, splitting pain in larynx, so severe as to compel coughing from the pain and to try to suppress cough; worse afternoon, evening, and in warm room: *All. c.*[2]

— — — — spasmodic whooping cough, in two paroxysms, occurring in quick succession, followed by a longer interval; excited by tickling in larynx, as if from down; worse afternoon to midnight; expectoration morning and day: Sulph.[2]

**SPASMODIC** cough, **tickling** creeping, occasional severe paroxysms of spasmodic cough, ejecting viscid yellowish mucus from bronchi, often flying forcibly out of mouth, caused by tickling as from sugar dissolving in larynx, and ending with sneezing and coryza: Bad.[2]

— — — — spasmodic cough in frequent paroxysms, with violent, shattering, hollow coughs, which follow each other in quick succession and do not allow one to recover breath, excited by tickling in upper larynx as if from vapor of sulphur; worse at night; morning expectoration: *Ipec.*[2]

— — — — spasmodic cough excited by intolerable tickling in larynx and in supra-sternal fossa; worse in morning and by vexation; evening expectoration: Iod.[2]

— — — — spasmodic cough, resembling whooping cough, the coughs follow each other in rapid succession, excited by tickling in chest from larynx to stomach; worse forenoon and evening till midnight: Sepia.[2]

— — — — compare with tickling in trachea.

— — **lying**, dry, hacking or spasmodic cough, worse lying, better sitting up; worse at night, after eating, drinking, talking, or singing: *Hyos.*[7]

— — **midnight**, agg. after: Dig., *Kali c.*, *Hyos.*, Squil.—2.

— — — — afternoon until: Mosch., *Sulph.*—2.

— — — — before: Mur. ac., Rhus, Sabad.—2.

— — — — evening until: Bar., Carbo v., Ferr., Led., Mez., Nitr. ac., *Puls.*, Rhus, Sepia, Stann., Zinc.—2.

**SPASMODIC** cough, **midnight,** agg. evening until after: Mag. c., Mag. m.—2.

— — **morning** agg.: Carbo v., Dig., Iod., Ipec., Kali c., Kreos., Ph. ac., Natr. m., Puls., Squil., Sulph.—12 and 2.

— — **night** agg.: Agar.,[7] Aur.,[7] *Bell.*, Bry., Calc., Con., Coral., Hyos., Ipec., Kali c., Mag. c.,[12] Mag. m.,[12] *Phos.*, Sil.—2.

— — — and day: Sulph.[12]

— — **pharynx**, ungovernable spasmodic cough, which seems as if it would burst the thorax, excited by a peculiar tickling in pharynx, which, again, is induced by a sensation of smothering in throat: Lact. v.[2]

— — **rough**, croaking, with danger of suffocation: Acon.[7]

— — **stomach**, frequent paroxysms of spasmodic hacking cough, excited by tickling in stomach, rarely so in larynx, worse day and in evening; expectoration morning and day: Lach.[2]

— — — paroxysms of spasmodic cough, resembling whooping cough, the coughs follow each other in rapid succession, excited by tickling in chest, from larynx to stomach; worse forenoon and evening until midnight: Sepia.[2]

— — **stomach, pit of,** spasmodic cough with painful contraction at pit of stomach: Ferr. acet.[6]

— — — — spasmodic cough like whooping cough, excited by roughness and tickling in throat and in epigastrium; generally worse evening till midnight; morning expectoration: Bar.[2]

— — — — shattering, barking spasmodic cough, excited by tickling in larynx and in epigastrium; worse evening until midnight, rarely only in daytime; morning and day expectoration: Nitr. ac.[2]

**SPASMODIC**—(*Continued*).

— — — — spasmodic whooping cough, excited by tickling in throat and in epigastrium; worse evening after lying down; morning expectoration; Natr. m.[2]

— — — — spasmodic whooping cough, as if from vapor of sulphur, or excited by tickling in throat and in epigastrium; worse evening and night; expectoration morning and day: Bry.[2]

— — **throat**, from irritation in throat of hot eructations: Lac. ac.

— — — creeping **irritation**, spasmodic hollow whooping cough, in short hard coughs and infrequent paroxysms, excited by feeling as if sulphur vapor were inhaled, or by creeping irritation in larynx and throat; worse evening till midnight; morning expectoration: Carbo v.[2]

— — — **roughness**, spasmodic cough, like whooping cough, excited by roughness and scraping in throat and in epigastrium; worse evening till midnight; expectoration in morning: Bar.[2]

— — — — see Scratching.

— — — **scraping**, see Roughness.

— — — **scratching**, hollow or whistling spasmodic cough, excited by roughness, scratching and tickling in chest and throat; worse morning and evening: Kreos.[2]

— — — **smothering** sensation, ungovernable spasmodic cough, which seems as if it would burst the thorax, excited by a peculiar tickling in pharynx, which again is induced by a sensation of smothering in throat: Lach. v.[2]

— — — **tickling**, asthmatic, hacking spasmodic cough, in short

**SPASMODIC**—(*Continued*).

but frequently recurring paroxysms, excited by tickling in throat and larynx; worse at night, especially after midnight; expectoration morning early and during day: Kali c.[2]

— — — spasmodic cough in old people at night, from continuous tickling in throat, as if palate were too long: Hyos.[12]

— — — — attacks of spasmodic cough, excited by tickling in throat; worse evening till after midnight; expectoration during day: Mag. m.[2]

— — — — spasmodic cough at night, occurring in paroxysms every quarter of an hour, each paroxysm consisting of but few coughs, with a rough, hollow, barking tone, excited by tickling in throat, as if from down or, as it were, by constriction of larynx; worse evening and night: Bell.[2]

— — — — short, spasmodic cough, in brief, but frequently repeated paroxysms, excited by tickling as if from feathers or down in throat and trachea; worse evening and night; expectoration in morning and during day: Calc.[2]

— — — — spasmodic whooping cough, excited by tickling in throat and epigastrium; worse evening after lying down, less in morning; morning expectoration: Natr. m.[2]

— — — — powerful spasmodic, nocturnal paroxysms of whooping cough, excited by itching and tickling in chest and throat, as if by a valve in larynx; worse at night; expectoration in day: Con.[2]

— — — — spasmodic whooping cough, as if from vapor of sul-

**SPASMODIC**—(*Continued*).

phur, or excited by tickling in throat and epigastrium; worse evening and night; expectoration morning and day: Bry.[2]

— — **trachea**: feeling as of constriction of chest and trachea, as if from sulphur vapor, excites spasmodic cough; worse from midday till toward midnight: Mosch.[2]

— — — **itching**, scratching in, spasmodic, shattering, whooping cough, often in paroxysms of two coughs each, excited by itching scratching with a feeling of dryness and, as it were, of vapor of sulphur in trachea and in chest; worse evening till midnight; expectoration morning and day: Puls.[2]

— — — **mucus** in, spasmodic cough in violent short paroxysms, excited by mucus in trachea and by creeping in chest; worse after midnight and in morning; difficult expectoration in morning: Squil.[2]

— — — — shattering spasmodic whooping cough, with frequent, rapidly succeeding coughs, excited by tickling as if from mucus firmly seated in trachea; worse at night, especially after midnight; day expectoration: Hyos.[2]

— — — **roughness** in, hollow, deep, spasmodic cough, excited by roughness and scratching in roof of mouth and in trachea; worse about midnight and morning; evening expectoration: Dig.[2]

— — — **scratching** in, hollow, deep, spasmodic cough, excited by roughness and scratching in roof of mouth and in trachea; worse about midnight and morn-

**SPASMODIC**—(*Continued*).
ing, cold fluids; evening expectoration: Dig.[2]

— — — **tickling** in, attacks of hollow, spasmodic cough, excited by tickling in larynx and trachea; expectoration chiefly at night: Staph.[2]

— — — — spasmodic cough, which stops breathing, produced by tickling deep in trachea; the cough is violent, dry, and hollow, and is agg. by stooping forward: Spig. (Dunham.)

— — — — exhausting, spasmodic cough, excited by tickling in larynx and in trachea as far down as middle of chest; worse afternoon and evening till midnight; expectoration morning and during day: Zinc.[2]

— — — — frequently recurring, whistling, spasmodic cough, in single coughs, excited by tickling in larynx and trachea, as if they were dry; worse in day especially toward evening: Laur.[2]

— — — — spasmodic cough, excited by tickling in larynx, in trachea, and in thyroid region; worse evening till beyond midnight; expectoration morning and day: Magn. c.[2]

— — — — short, spasmodic cough, in brief, but frequent paroxysms, excited by tickling as if from feathers or down in throat and trachea; worse evening and night; morning and day expectoration: Calc.[2]

— — — — spasmodic cough, excited by tickling in trachea; worse in evening till midnight; during this period sputa cannot be dislodged (at night must sit up to raise the sputa[7]), but in day,

**SPASMODIC**—(*Continued*).
during motion, they become loosened: Ferr.[2]

— — — — spasmodic, shattering cough, excited by tickling creeping in larynx and trachea and chest; worse evening till midnight; no expectoration in evening: Rhus.[2]

— — violent, spasmodic cough, with frequent eructations and hoarseness: *Ambra.*[7]

— — violent, spasmodic cough at night: Agar.[7]

— — with **vomiting**: Bry., Carbo v., Ferr., Ipec., Puls.—12.

— — **women**, spasmodic cough, dry, nervous, peculiar to women; periodically, every night from sunset to sunrise: Aurum.[7]

**SPASM**, whooping cough, children get stiff, before coughing the child raises herself suddenly, looks wildly about, the whole body becomes stiff, loses consciousness, first as if she would have an epileptic spasm, then follows the cough: Cina.

— whooping cough, children get stiff, respiration ceases, spasmodic twitchings, then consciousness returns; they vomit and recover slowly: Cupr.[7]

— with cough: Cham., *Cina*, Croc., Cupr.,[5] Dros., Hyos., Led.,[5] Meph.,[5] Verat.—16.

**SPEAK**, every attempt to, excites cough, so that one must desist: *Cimic.*[14]

— **cannot**, a word for uninterrupted cough, with discharge of bloody mucus from nose, after seawind: *Cupr.*[7]

— — cough of such a nature as not to allow the utterance of an audible word: *Merc.*[14]

**SPEAKING** (talking) agg. or excites cough: Acon., Ambr., Anac., Arn., Ars.,[1] Bar., Bell., Bry., Calad.,[1] Calc., Carbo v., *Caust.*,[1] Cham., *China*,[11] *Cimic.*, *Cupr.*,[7] Dig., Dulc., Erio.,[1] *Euphr.*, *Hepar*, Ign., Iod., Ipec., *Lach.*,[11] Mag. c., Mag. m., Mang., Meph., *Merc.*, Mez., Mur. ac., Natr. m.,[11] Nitr. ac., Phos., Ph. ac., Psor.,[1] Rhus, Rumex, Sil., Spong., Squil., *Stann.*, Stram., Sulph., Sul. ac., Verb.—5.
— **convulsive** cough: Dig.
— **dry** cough: Atro., Dig., *Mang.*, Ment. p.
— **hacking** cough, morning: Sum.
— **larynx**, causes tickling in and cough: *Alumen.*
— **loud**, causes cough: *Coc. c.*
— **periodic** cough from smoking or: Atro.
— **short** cough: Ant. t.
— **tickling** cough on: Alumen.
— **violent** cough, agg. by: Mur. ac.
— **whooping** cough when: Anac.

**SPELLS** of coughing. See Paroxysmal Cough.

**SPIRITS**, drinking, agg. cough: Arn., Ferr., Ign., Lach., Led., Spong., Stann., Stram., Zinc.—5.

**SPLITTING** cough, night: Aur.
— See Head, Chest, etc.

**SPOKEN to**, cough on being: Ars.

**SPRING**, cough in the: Ambra, Verat.—2.

**STANDING**, agg. cough: Euphr., Natr. s., Sepia, Sulph., Zinc.—5.
— **breathing**, frequent cough, with some sputum, stitches in left side of chest, short breathing if coughing while standing: Natr. s.[7]
— **erect**, cough while: Acon., Natr. m.,[5] Stann.—12.
— **still** (stopping) during a walk; agg. cough: Ign.

**STARTING**, cough on starting in sleep: Apis.
— in sleep, with: Cina, Hepar.—12.
— from sleep with cough; drinking relieves it: Brom.[7]

**STAIRS**, ascending, agg. cough: Arg. n.,[7] *Ars.*,[1] Bar., Iod., Lyc.,[1] Mag. c., Mag. m., Merc., Nitrum, Nux v., Seneg., Sep., Spong., Squil., Stann., Staph., Zinc.—5.

**STERNUM**, **aching** behind, on sitting bent forward, morning, causing short cough: Rhus.
— **aching** in middle of, when coughing: Nitrum.
— **aching** in upper part, with catarrh and cough: Ferr. acet.[6]
— **burning** pain under the, and stitches in sides of lungs, with hacking cough: Clem.[10]
— **burning** in upper part, after cough: Ferr.[6]
— **constrictive** sensation under, during cough great oppression of chest, so that patient must sit up in bed, and in order to expectorate, when she experiences great pain with: PHOS.
— **dust**, cough with sensation as of dust under sternum, after waking and on rising: *Chelid.*
— **excoriation**, pain as from, in upper part, on coughing: Hydras.
— **fly to pieces**, feels as if it would, from violent cough, feels sore when talking, laughing, or yawning: Mur. ac.[6]
— **hand** presses on, during cough: Bry.[17]
— **irritation** in mid-, excites dry incessant cough: Mang.[7]
— **irritation** and tickling behind, after siesta, cough from: Rhus.
— **irritation** in upper part causing cough: Natr. ars., Cham., Plat.,[14] *Rhus.*

**STERNUM**, **irritation** under, when coughing: Arg. n.[7]
— **itching** under middle of, excites cough: Nux v.
— **pain** behind, when coughing: Chelid., Cori. m., Staph., Thuja.
— **pain** behind, going into side on coughing: Osm.
— **pain** in, with the cough: Amm. c., Bell., China, Mez., Sep., Sil. —12.
— **pain** in lower part of, from cough: Amm. c., Phos.
— **pain** in lower part of, 8 A. M., from cough: Dios.
— **pain** from mid-sternum through to back with cough (raises tough black mucus[6]): *Kali b.*
— **pain** in upper part of, when coughing: *Sang.*, Sep.
— **pain** in upper part of, on coughing, morning: Sil.
— **presses** hand on, during cough: Bry.[17]
— **pressure**, as if it would be pressed out; worse from pressure, or stooping or coughing: Ph. ac.[6]
— **pressure** beneath, after coughing: *Euphr.*
— **pressure** from middle part of, into larynx, during cough: Kali b.
— **pressure** in middle of lower portion of, excites cough: Cimex.
— **pressure** from, to back, sensation as of a stone pressing down in pleural cavities, causing painful cough with: Coral.[6]
— **rawness** behind, cough from: Nitrum.
— **rawness**, scraping behind, cough from: Kreos.
— **scraping** at lower part of, and stitches in right frontal eminence. Mez.[6]
— **scraping**, rawness behind, cough from: Kreos.

**STERNUM**, **scratching**, rough cough scratching the breast immediately under: Cann. ind.
— **soreness** behind, on coughing: Amm. c., Osm.
— **soreness**, behind upper part of on coughing: Chel.
— **stifling** sensation beneath upper fourth of, hysterical cough from a: Plat.[7]
— **stitches** in, on coughing: Bell., BRY.
— — — when coughing, obliging him to hold chest with the hand; stitches also felt when feeling the parts: Bry.[6]
— **stitches** in ensiform cartilage when coughing: Sulph.
— — in middle part of, on coughing: Sil.
— **stitching** pain from behind, short dry cough, with deep: Berb.[6]
— **tearing**, extending from middle of, to throat, on coughing: Psor.
— **tickling** behind, extending to back, with irritation to cough: August.
— **tickling** behind upper half of, when sitting bent forward, 11 A. M., dry cough: *Rhus.*
— **tickling** behind upper, causes short, 11 P. M.: Rhus.
— **tickling** and irritation behind upper half of, short cough from severe, followed by a feeling of discouragement and apprehension: RHUS.
— **tickling** irritation to cough under manubrium of: Cina.
— **tickling** under, causes cough: Thuja, Zinc.
— **tickling** under lower part of, causing cough: Verat.
— **tickling** under middle of, causing cough: Ang., Con., Thuja.

**STERNUM, tickling** under upper part of, causing cough: Rhus, Rhus r.,[14] Rumex.
— **tickling** under upper part of, causing distressing cough: Ir. v.
— **tickling** sensation under upper half of, exciting short, dry cough, with dull aching in left mammary region, when sitting inclined forward, at 11 A. M.: *Rhus.*
— **torn loose**, sensation as if something had been, under mid-sternum, cough with transparent expectoration: *Phos.*[6]
— **torn off**, soreness in upper part under, from morning cough; after expectoration this part continues to feel sore and burning, as if something had been: Cina.[6]

**STERTOROUS** cough, agg. at night: Cact.
— breathing with cough: Bell.[5]

**STIFF**, children get stiff, breathing ceases, spasmodic twitchings, after awhile consciousness returns, they vomit and recover, but slowly in whooping cough: Cupr.[7]
— before coughing the child raises herself suddenly, looks wildly about, the whole body becomes stiff, she loses consciousness, just as if she would have an epileptic spasm, then follows the cough: Cina.
— suffocative cough, whereby child gets quite stiff and blue in the face: IPEC.

**STIFFNESS** (rigidity) of body, with cough: Bell., Cina, Cupr., Ipec., Led., Mosch.—2.

**STIMULANTS**. See Spirits.

**STITCHES**. See under Chest, etc., etc.

**STOMACH** and diaphragm affected by night cough, mostly before sunset: LYC.

**STOMACH, cold feeling** in, with dry suffocative cough: Lact.[10]
— **comes** from, cough seems to come from the: All. s.,[1] Ant. t., Bell., Bry., Ery. m.,[1] Merc., Puls., SEPIA.—5.
— — croaking cough, comes from stomach in paroxysms, during day: Nitr. ac.
— — short dry cough, which seems to come from the: SEPIA.
— **contraction** in stomach and pit of after coughing: Ars.
— **cutting** in, with cough: Verat.[5]
— **gnawing** in; better from food: Grat., Natr. c.—5.
— **gnawing** feeling of coldness in stomach and in pit of, and sleeplessness, from dry suffocative cough, in hysterical female: Lact.[6]
— **heaving** and swelling at, before the cough: Kali. b.[6]
— **irritation** in, excites cough: Bell., Bor.,[12] Bry., Merc., Puls.,[12] Sep.[12]—5.
— **irritation** as if from stomach, with cough, for many nights; waking or sleeping: *Merc. sol.*
— **jerks** in, with cough: Ipec.[12]
— **pain** in, from cough: Bell., Bry., Hell., Ipec., Lyc., Nitr. ac.,[12] Phos., Puls., Rhus, Rumex, Ruta, Sabad., Sepia.—5.
— — — before an attack of coughing: Ant. t., Arn., Bell., Cham. —12.
— — dry cough, with perspiration, and water in the eyes; with stitches in the vertex, vomiting and pain in stomach: Sabad.[10]
— — dry teasing cough, caused by tickling in bronchia, or even uncovering a hand, with tearing pain in chest, stitches and profuse sweat and pain in stomach: Rhus.[7]

**STOMACH PIT** (epigastric region), **beating** pain in head and pit of stomach after coughing: Ipec.[6]

— — **bruised** pain in, from coughing: NUX V., STANN.,[1] Squil.—6.

— — **coldness**, gnawing feeling of, in stomach and in pit of, and sleeplessness, from dry suffocative cough, in a hysterical female: Lact.[6]

— — **coming** from, croaking cough coming from pit of stomach, by paroxysms, during day: Nitr. ac.

— — **contraction**, painful, in, preceding the cough: Ferr. acet.[6]

— — **contractive** pain in, causes cough to continue (even after sitting up): Ars.[7]

— — **crawling** about pit of stomach, extending to throat, excites hacking cough: Plat.

— — **crawling** tickling in, dry cough as if coming out from stomach with: *Bry.*

— — **emptiness** in, sensation of, with cough: Croc.,[5] Ign., Mur. ac., Stann.[2]

— — **irritation** to cough, felt in: Bar., Bell.,[5] Bry., Cham., Guai.,[12] Hepar, Ign.,[16] Lach., Merc.,[5] Natr. m., Nitr. ac., Ph. ac., Puls.[5]—2.

— — **irritation** at, causes troublesome, usually dry cough: Ign.[14]

— — **itching**, cough caused by itching in trachea, extending from pit of stomach to epiglottis: *Puls.*

— — **laying** (pressing) hand on, amel. dry, or exhausting or paroxysmal cough: CROC.

— — **oppression** in, excites cough: Kali b.[2]

— — **pain**, beating, in head and pit of stomach after coughing: Ipec.[6]

**STOMACH PIT, pain, bruised,** in, from coughing: NUX V., STANN.,[1] Squil.—6.

— — **pain** in, from coughing: Alum., Ambra,[5] Amm. c., *Ars.*, Bry., DROS., Lach., Mang., NUX V., Sepia, Silicea, Thuja.—12.

— — — painful contraction in pit of stomach (early morning in bed), followed by a kind of spasmodic cough with expectoration of transparent and tenacious mucus: Ferr. acet.[6]

— — — continued dry cough attended by vomiting and arrest of breathing, and lancinating pain, extending from left side of abdomen to hypochondria and pit of stomach: Alum.[6]

— — — dry cough in evening, with pains in pit of stomach: Arundo.

— — **pressure** on, amel. cough: Croc.[5]

— — — — amel. pain of cough: *Dros.*

— — — in, excites cough: Calad.[5]

— — — — with cough: Phos.[5]

— — **roughness** and scraping in throat and in epigastrium, excites spasmodic cough, like whooping cough; worse evening till midnight, from warm food and in company of strangers: *Bar.*[2]

— — **something** in, sensation as if, excites cough: Bell.[7]

— — — cough; it seems as if something came from the epigastrium; so also when she laughs; there is a good deal of tickling in larynx, nevertheless it seems to start from the epigastrium: Raph.

— — **small spot** in, painful to touch, violent cough appears to come from: Kali b.[6]

— — **soreness** in, from cough: Bry.,[6] *Nux v.*[10]

**STOMACH PIT**, **sticking** in, with cough: Tabac.[5]
— — **stitches** in, with cough: Ars., Bry., Kali b., Phos., Sulph. —5.
— — **tender spot** in, seems to cause cough: Kali b.[6]
— — **tickling** in, excites cough: Bar., Bell., Bry., Guai., Hepar, Lach., Natr. m., Nitr. ac., Ph. ac. —5.
— — — dry cough from tickling low down in chest, just above pit of stomach; cough is worse evenings after lying down: PH. AC.
— — — shattering, barking, spasmodic cough, excited by tickling in larynx and in epigastrium; worse evening until midnight: Nitr. ac.[2]
— — — spasmodic whooping cough, excited by tickling in throat and in epigastrium; worse evening after lying down: Natr. m.[2]
— — — spasmodic whooping cough, as if from vapor of sulphur, or excited by tickling in throat and in epigastrium, worse evening and night: Bry.[2]

**STOMACH re-echo**, cough seems to, in stomach: Cupr.
— **dry** cough, as if proceeding from stomach or abdomen, or from constipation, or as if something remained in stomach that would not pass off: SEPIA.
— **sinking** at: Hepar, D5.ig.—
— **struck**, feels as if struck by the cough: Bell.[5]
— **swelling** and heaving at, before the cough: Kali b.[6]
— **tickling** in, excites cough: Bry.,[5] Sang.[1]
— — — with cough: Rumex.[5]
— — paroxysms of spasmodic cough, resembling whooping cough, the coughs following each other in rapid succession, excited by tickling in chest from larynx to stomach; worse forenoon and evening till midnight: Sepia.[2]
— — frequent paroxysms of hacking spasmodic cough, excited by tickling in stomach, rarely, also in larynx; worse in daytime and in evening: Lach.[2]
— **turned** inside out, dry severe cough, mostly in morning, with retching and desire to vomit, and sensation as if stomach were turned inside out: *Puls.*[10]
— **weakness** of, great, from cough with expectoration: Lyc.[6]

**STOPPAGE** of breath. See Dyspnœa.

**STOOLS**, **frequent** stools amel. dry cough: Bufo.
— **involuntary**, when coughing: Phos.,[7] *Verat.*[10]
— compare with Diarrhœa.

**STOOPING**, excites coughing: Arn., Bar., Caust., Chel., Kali c., Laur., Lyc., Phos., Senega, Sil., Spig.,[1] Spong., Staph., Verat.—12.
— agg. dry or hollow cough: Spig.
— after rising from: Phos.

**STRAINING** cough: Asp., *Cupr.*, Thuja.

**STRANGERS**, child coughs at sight of: Ambra,[8] Bar.[2]

**STRETCHING** out arm excites cough: Lyc.[2]

**STUFFING** cough with pain at chest and expectoration of yellowish, green, tough matter: Kali b.

**SUCKLING**, cough while nursing the child, with expectoration of blood: Ferr.

**SUDDEN** cough: *Agar.*, Am. bro., *Euphr.*, Kali c., Naja.

— — day: Coloc.

**SUDDEN** cough, evening: Apoc. c.
— — — tickling in throat: *Am. bro.*
— — **irritation** to: Kali b., *Sepia.*
— — — from tickling below tonsils: Am. bro.
— — morning: SQUIL.
— — night: Apoc. c.
— — tickling in throat, evening: Am. bro.
— — cough comes so suddenly and is so violent that it cannot be suppressed; several shocks succeed one another, causing him to double up, and force tears from his eyes: Agar.

**SUGAR**, eating, amel. cough: Sulph.[5]
— — cough from: Zinc.[5]

**SUFFOCATION** with cough: compare with Interrupted Respiration, Difficult Breathing, etc.
— suffocative arrest of breathing before cough: Bry., Coral., Led.[5]
— inclination to cough is so sudden and violent, can scarcely inhale at all: *Sepia.*[7]
— complete suffocative feeling, cannot exhale, with whooping cough: Meph.[10]
— cough in long continued attacks causing suffocative fits; a swallow of cold water relieves cough: Cupr.[10]
— painfulness of larynx, with danger of suffocation, when touching or turning neck, coughing, or drawing a long breath: Bell.[6]
— as soon as one falls into a sound sleep, has a choking cough as if he would suffocate: LACH.[14]
— cough threatening suffocation, with spasmodic contraction of larynx, every effort at inspiration increases the cough: *Meng.*[14]
— on swallowing a sudden paroxysm of cough with suffocation: BROM.

**SUFFOCATIVE** (choking) cough: Acon., Anac., *Ant. t.*, Aqua. petr.,[1] Ars., Bar., Brom., *Bry.*, *Carbo an.*, *Carbo v.*, *Cham.*, CHINA, CINA, Cocc., Cocc. c., *Con.*, CUPR., Cycl., Delph.,[1] Der.,[1] DROS., Euphr., Eupion,[1] Guare.,[1] *Hepar*, Indigo, IPEC., Kreos., *Lach.*, Lact., *Led.*, Mang., Natr. m., *Nux m.*, NUX V., *Op.*, Petr., Phell.,[12] *Puls.*, SAMB., *Sep.*, Sil., Spig., Spong., Squil., SULPH., *Tabac.*,[12] Tep.,[1] Zinc.[1]—5 and 11.
— — **bed**, a violent cough inducing vomiting, or a suffocative cough before and after going to bed, also at night: Indigo.[6]
— — **chest**, suffocative cough with expectoration of much tough mucus, which accumulates in chest and throat and is difficult to raise, causing almost strangulation, and vomiting of food; worse night after going to bed and in morning while lying down: Coc. c.[2]
— — — suffocative, hollow, deep, whooping cough, excited by spasm of chest; worse about or just after midnight: Samb.[2]
— — — hollow, suffocating cough, resembling whooping cough, provoked by tickling in chest, throat, larynx, and suprasternal fossa; worse at night and from anger: Cham.[2]
— — **day**: Anac.
— — — 2 and 4 A. M.: China.
— — — 5 A. M., dryness in larynx: *Kali c.*
— — in **children**: Bry., *Samb.*, *Sulph.*—12.
— — — — with crying and weeping: Samb.[12]
— — after **eating** and drinking: Bry.[12]

**SUFFOCATIVE cough evening**: *Carbo an.*, Indigo,[1] *Ipec.*,[1] Natr. m.—12.

— — — 7 P. M.: *Ipec.*

— — — after going to sleep: Carbo an.,[1] *Lach.*[14]

— — whereby the child becomes quite stiff and blue in the face: IPEC.

— — **larynx**, from dryness in, 5 A. M.: *Kali c.*

— — — suffocative, hoarse cough, excited by dryness and rawness in larynx and trachea; worse evening and night, and lying on right side: Carbo an.

— — — suffocative, hollow cough, resembling whooping cough, provoked by tickling in chest, larynx, throat, and suprasternal fossa; worse at night, and from anger: Cham.[2]

— — — suffocative cough as when water gets in larynx: Spig.[6]

— — **night**: *Ars.*, Bell.

— — — dry suffocative cough, worse at night: Cupr.[7]

— — **noon**: *Arg. n.*[7]

— — **respiration**, from tightness of: *Hepar.*

— — **sleep**, after going to, evening: Carbo an.,[1] *Lach.*[14]

— — **stiff**, suffocative cough whereby child becomes quite stiff and blue in face: *Ipec.*

— — **stomach**, dry, suffocative cough, in a hysterical female, with gnawing feeling of coldness in stomach and in pit of stomach and sleeplessness: Lact.[6]

— — **throat**, suffocative, hollow cough, resembling whooping cough, provoked by tickling in chest, throat, and suprasternal fossa; worse at night and from anger: Cham.[2]

— — — from tickling in: *Hepar.*

**SUFFOCATIVE** (choking) **trachea**, from dryness and scraping in, in open air: Cycl.

— — — suffocative, hoarse cough, excited by rawness and dryness in larynx and trachea; worse evening during sleep, and lying on right side: Carbo an.[2]

— — — dry suffocative cough, caused by scraping and dryness in trachea: Cyc.[7]

— **fits** with the cough: Acon., *Ant. t.*, Ars., Bry., Cham., Coral., *Cupr.*, *Dros.*, Hepar, *Ipec.*, Lach., *Led.*, Meny., *Meph.*, Mosch., Nicc., Nux v.,[6] Op., Spig., Spong. —5.

— — compare with Interrupted Respiration.

**SULPHUR fumes**, or vapor, sensation of, excites cough: Amm. c., Amyl. n.,[1] ARS., Asaf.,[5] Brom., Bry., Calc., Carbo v., *China*, Cina, IGN., Ipec.,[5] Kali chl.,[5] *Lach.*, *Lyc.*, Mosch.,[12] *Par.*, PULS.—11.

— — laryngeal cough, from sensation of, in upper part of larynx: *Ars.*

— — tickling cough from sensation of, in larynx: *Lyc.*

— — sensation as from fumes of burning match in throat cause irritation to cough: Amyl. n.

**SUN**, cough agg. by heat of: *Ant., cr.*[5]

— walking in hot, paroxysmal cough: Coca.[1]

**SUPPER**, hacking cough after: Natr. ars.

**SUPPRESS**, uninterrupted provocation, in larynx, to a hacking cough; it does not disappear on coughing, but only when suppressing the cough: IGN.

— titillating sensation in evening after lying down, as if dust had been inhaled in trachea, inducing

**SUPPRESS**—(*Continued*).
a disagreeable, dry, irritating cough which cannot be suppressed and becomes worse as coughing continues: Marum.[6]
— great desire to cough, but it is partially suppressed; it does not amount to a full, free cough: Sulph.[14]

**SUPRASTERNAL** fossa. See Throat Pit.

**SURPRISE**, happy, causes weeping, coughing, and trembling: Acon., Merc.[8]

**SWALLOWING agg.** or excites cough: *Brom.*,[1] Eugen.,[1] Kali hypermang.,[1] Opium, Phos., Puls.—5.
— **amel.** irritation to cough: Eugen.[5]
— — continued swallowing relieves soreness and cough: Apis.[7]
— — swallowing mucus, amel. paroxysmal cough, before midnight: Apis.
— — compare with Drinking.
— **empty**, irritation to cough from: Lyc., *Natr. m.*
— **hacking** cough from: Kali hypermang.
— — — followed immediately by an effort to swallow something: *Cina.*[7]
— **short** cough, agg. by: *Aesc. h.*
— cough in long continued attacks causing suffocative fits, a swallow of water relieves: *Cupr.*[10]
— on swallowing, a sudden paroxysm of cough with suffocation: BROM.

**SWEAT** profuse, it stands out on face in beads; stitches in sides and cough: Arg. n.[7]
— profuse on whole body with cough: Opium.[7]

**SWEAT** over whole body, which brought on a cough, with violent retching: Dros.[7]
— See Perspiration.

**SWEETMEATS**, cough from: Zinc.[2]

**TASTE bad** after the cough: Cocc.[5]
— **bloody**, when coughing: Amm. c., Bell., Elaps, Kali b., Nitr. ac., Rhus.—5.
— **offensive**, with cough: *Caps.*
— **sour**, when with cough: Cocc.
— **sweet** with cough: Aeth., Amm. c.,[10] Canc. f.—1.
— — cough with spitting of blood, with previous sweet taste and great dyspnœa: Amm. c.[10]
— — sweetish taste in throat, while sitting forenoon with cough: Mag. c.

**TEA** agg. cough: Ferr., Spong.—5.
— **hot**, cough agg. after: Spong.[6]

**TEASING** cough: Coloc., Linum, Meli., Phos.
— tickling cough and frothy mucous expectoration, when out in open air: Linum.

**TEETH**, cleaning the teeth provoked a violent cough, with vomiting of slimy fluid: *Coc. c.*
— grinding of the: Bell., Podo.[5]
— — violent cough during sleep with grinding of the teeth: *Bell.*

**TEETHING**, cough during dentition: Calc., Calc. ph.,[7] *Cham.*, Cina, Hyos., Rhus.—5.
— dentition tardy and often attended by convulsions and a loose rattling cough: *Calc.*[22]

**TEMPERATURE**, a change of, excites dry cough: *Acon.*
— — — compare with going into open air, or entering warm room, etc.

**TEMPLE, aching** in, agg. by cough: Tax.

**TEMPLE, burst**, sensation as if temple would burst, when coughing: Cina.[6]
— **pain** in, with cough: Ambra, Caust., Cina, Kali c., Kreos., Lyc., Puls., Rhus, Verb.—2.
— — in right temple, from cough: Mang.[1]
— **pain** in, and on top of head from coughing: Alum.[7]
— **shooting** in, when coughing: Coca.
— **stitches** in, from cough: Caust., Cina, Kali c.—2.
— **tearing** in, from coughing: Alum., Puls.—2.
— **tension** in, with the cough: Verb.[2]
— compare with head, sides of.
**TENACIOUS** cough: Ars.
**TESTICLES**, pain in, when coughing: Zinc.[5]
**THIGH**, deep pain in thigh extending to knee when coughing: *Caps.*[6]
**THINKING**, cough worse when thinking of it: Bar., *Ox. acid.*—8.
**THICK** cough: Aur., Aur. s.
— — at 6 P. M.: Chelid.
— — agg. by bad weather: Aur. s.
**THIRST**, cough accompanied by: Acon., Ant. t., Ars., Bry., Cocc. c., Samb.—5.
— for large quantities seldom: Bry.[5]
— for small quantities, frequently: Ant. t., Ars.—5.
**THROAT, aching** in, from cough: Coc. c.
— **acrid** sensation in, with cough: *Nux v.*[5]
— **burning** in, after cough: Atropia, Coc. c., *Mur. ac.*, Phos.,[5] Ph. ac.[6]—1.
— — dryness in, with cough: Lact.[6]
— — — tickling cough with very dry throat: SANG.

**THROAT, burning** in, excites cough: Urt. ur.
— — — — dry cough: Bov., Kali b.
— — — — hacking cough: Kali b.
— — — — irritation to cough: Acon. f.
— **burning** soreness in, with cough: Sulph.[5]
— **catarrh**, sneezing with catarrh in throat that provokes cough: Petro.
— **clear**, desire to clear the throat, with rough feeling in the throat as if mucus were adhering to it, with titallation inducing cough: Lyc.[6]
— **constriction** of, causing cough: Coc. c.,[5] Ign.[1]
— — in, with cough: Asar.[5]
— — cough from constriction in throat during sleep, at night: Nitr. ac.
— — lower down in throat excites cough: Lac. ac.
— — as from sulphur fumes in pit of throat excites cough: Ign.
— **contraction** of, from the cough: Ars., Lach.—12.
— — choking contraction in upper part of throat, cough from: Cocc.
— — of skin of, causing cough: Zing.
— — cough from contractive titallating sensation in throat, from larynx to lowest bronchial tubes: *Ipec.*[6]
— **crawling** in, cough from: Amm. m., Bry., Carbo v., *Kali c.*, Kreos., Stann.
— — cough from a constant crawling upward in: *Bry.*
— — hacking cough from: Colch., Euph., Lach., Prun. sp.
— — involuntary half cough from roughness and crawling in: *Carbo v.*

**THROAT, crawling** sensation in throat, below larynx, causes a dry scraping cough: *Kreos.*[14]

— — sensation in ulcer in throat, excites short cough: *Lach.*

— — and tickling in throat and pit of, excites cough: Sang.

— — — — —, excites dry cough: Calc. ph.

— — **upward** in, cough from constant crawling: *Bry.*

— **creeping** cough excited by creeping sensation from chest to throat, particularly during pregnancy: Nux m.[14]

— — spasmodic hollow whooping cough in short hard coughs and infrequent paroxysms, excited by feeling as if sulphur vapor were inhaled; or by creeping irritation in larynx and throat; worse evening till midnight: Carbo v.[2]

— — short, hollow, unceasing cough excited by creeping tickling and by much mucus in throat; worse evening till midnight: Caust.[2]

— **crumb**, feeling as of a crumb in throat excites cough: *Lach.*[5]

— **cutting** in, when coughing: Sulph.

— **draught** of air into, excites irritation to cough: Plan.

— **down** in, see Feather, sensation as of.

— **dry** spot in, causes cough: *Cimic.*[7]

— — — — larynx causes cough: Con.

— **dryness** in, agg. coughing: Bry., Carbo an.,[12] Carbon ox.,[1] Dros., Iodf.,[1] Lach.,[12] Lachn., Lauro.,[12] Mang., Petro., Phyt.,[21] Puls., Rhus, Sang.,[1] Senega, Stann., Sulph.[1]—5.

— — — **afternoon**, in the, causes dry, hacking or teasing cough: *Sang.*

**THROAT, dryness in, dry** cough in the: Plan., Stann.

— — — **hacking** cough: Plan.

— — — — — morning: Ant. t., Mang.

— — — **irritation** to cough: Atropia.

— — — **loud** cough: Stann.

— — — **morning**, hacking cough: Ant. t., Mang.

— — — **night**, cough at, causing dryness of throat: Puls.

— — — great dryness of throat causing cough with aching pains in throat: Kalmia.[7]

— — — **rasping** cough: Stram.

— — — **tickling** cough: Sang.

— — — **violent** cough: Kali chl.

— — — **with** cough: Eugen.,[1] Kali chl., Lachn., Merc.,[12] Osm., Phos., Puls., Squil., Rhus, Sulph.[1]—5.

— — — and burning with the cough: Phos.[12]

— **dust**, dry cough as from dust in throat, followed by nausea: Pic. ac.

— — dry cough as from dust in throat, agg. at night: *Amm. c.*

— — — — as from dust in pit of throat, agg. evening and by coughing: Ign.

— **feather** dust in, cough as from: *Amm. c.*,[5] *Bell.*, Brom.,[12] *Calc.*, Chelid., Cina, Dros., Hepar, Ign., Ph. ac., *Sulph.*—12.

— **filling** up, sensation as of, causes convulsive cough; night: Apis.

— — — — — — causes hoarse, hollow or convulsive cough: Apis.

— — — throat feels as if filled up, as if he could not swallow; frequent cough, bringing up white, frothy mucus: Silicea.[7]

— **flesh**, sensation as if cough would tear a piece of flesh from the throat: *Phos.*[6]

**THROAT**, **foreign body**, sensation of, in, causes cough: Am. caus.

— — — frequent short dry cough excited by tickling in larynx, with sensation of foreign body in throat which obstructs respiration: Lobelia.[2]

— **fruit**, when eating fruit feels as if something stuck in throat exciting cough: Arg.

— **full** feeling in, with cough: Cubeba.[5]

— **grasps** at throat, during coughing spell: Acon., All. c.

— **grease**, titillation in, as from rancid grease, with cough: Hepar.[6]

— **heat** in, after cough: Aur. m.

— **heat** in, frequent, paroxysms of dry, violent, hollow or short exhausting cough, excited by severe tickling in larynx, which brings tears to the eyes; by heat in trachea, and by sensation of dust in trachea, throat, and behind sternum, which is not relieved by coughing: *Chelid.*[2]

— **heaviness**, cough from heaviness in throat extending into chest: Iod.

— **inflamed** with the cough: Acon., Bell., Cham., Ipec., Lach., Nux v., Puls.—5.

— **irritation** in, excites cough: *Acon.*, *Ambr.*, Amm. c., Anac., Ant. cr., Ant. t., Aphis,[1] Ars., Asaf., Asar., Bar., Bell., Bov., Brom., Bry., *Calad.*, Carbo an., Carbo v., Caust., Cham., Chenop., China, Cocc., Coff.,[5] *Coloc.*, *Con.*, Croc., Dios.[1] (r.), Dros., Ferr., Graph., Hepar, Hura,[1] Hydr. ac., Hyos., Hyper.,[7] Kali c., Kali iod., Lach.,[1] Laur., Lith. c.,[7] Lyc.,[1] Mag. c., Mag. m., Marum, *Merc.*, Mez., *Natr. m.*, Nicc., Nitr. ac.,

**THROAT**, irritation in—(*Cont'ed*). Nux v., Ol. an., Par., Petro., Ph. ac., Plan.,[1] Puls., Rhod., Rhus, Sabad., Sars., Sepia, *Sil.*,[1] *Squil.*, Staph., Stront., Sulph.,[1] Sum.,[1] Tabac., Tabac.,[1] Tromb.,[1] Thuja,[1] Verat., Verb., Zinc.—12.

— **irritation** in, exciting dry cough: Aphis, Carb. ac., Lyc.,[6] Tabac., Thuja.

— — — **evening**, violent dry cough in evening, while lying down, compelling to rise; the irritation to cough is in a little spot posteriorly and inferiorly in throat: Lith. c.[10]

— — — **hacking** cough: *Sum.*, Thuja, Tromb.

— — — — —, much increased by heat and cold air: Hydr.

— — — when **hawking**, exciting cough: Raph.

— — — from **hot** eructations, cause convulsive cough: Lac. ac.

— **irritation** in, even unto choking with spasmodic cough, sore pain on top of head and subsequent debility: Kali c.[6]

— — — spasmodic hollow whooping cough in short hard coughs and infrequent paroxysms, excited by feeling as if sulphur vapor were inhaled, or by creeping irritation in larynx and throat; worse evening till midnight: Carbo v.[2]

— — —, causes **irritation** to cough: *Am. bro.*, Dios.

— — —, — — — — 10 P. M.: *Dios.*

— — — **low** down, when lying, excites cough: Lith. c.

— — — **moan**, sudden and involuntary; half cough and half a moan, caused by irritation in throat: Calad.[7]

**THROAT**, **irritation** in, **morning**, in left side, cough: Dios.
— — — **night**, cough from: Ambra.
— — — — convulsive cough from irritation in throat, as from hot eructations: Lac. ac.
— — — **short** cough: Carb. ac.
— — — **side** of, morning, cough: Dios. (l.)
— — — as from **smoke**, cough: Cocc.
— **irritation** to cough felt in: *Ambra*, Am. bro.,[1] Amm. c., Anac., Bov., Bry., Calc., Carbo an., Carbo v., Caust., Chenop., *Con.*, Graph., Kali c., Laur., Mag. c., Mag. m., Mang., Mez., *Natr. m.*, Nux v., Ol. an., Petr., Puls., Rhod., Sabad., Sars., Stront., Tabac.—12.
— — — —, deep in throat: Ambra, Aspar., Dios., Squil.
— — — — from draft of air: Plan.
— — — — — burning in: Acon. f.
— — — — — dryness in: Atro.
— — — — — irritation in: *Am. bro.*, Dios.
— — — — — irritation in, 10 P. M.: Dios.
— — — — from sensation as from fumes of burning match: Aml. n.
— — — — — sticking, griping: Mur. ac.
— — — — — sticking in throat: Phos.
— — — — on stretching it: Lyc.
— — — — felt in a little spot, inferiorly and posteriorly in throat: Lith. c.[10]
— — — — from tickling in: Asp., Natr. c.
— — — — in pit of throat: Ph. ac.
— — — — from tickling in throat, 9 P. M.: Dios.

**THROAT**, **itching** in, exciting cough: Calc. s.,[1] *Con.*, Guare.,[1] Nux v., Puls.—12.
— — — hacking cough, in morning: Ant. t., *Con.*
— — tickling deep in pit of throat, cough: Sil.
— — with coughing: Ambra.[5]
— **liquid** in, hacking cough, commencing in throat pit, a cool saltish liquid being lodged in the lower part of the throat: Cann. s.[6]
— **loose** skin hanging in, dry hacking cough from sensation of: Alum.[7]
— **mucus** in, excites coughing: Æsc. h., Am. cau., Arg. n.,[7] Atrop., *Arum t.*, Asar.,[7] Caust., Cham., Chin. s., Cina, Cocc., Coc. c.,[2] Euphr., Grat., Hyos., Kreos.,[1] Kali b.,[7] Laur., Phell., Plan., Raph.,[6] Seneg., Stram., Zinc.—1 and 5.
— — — on going to **bed**, cough: Iodf.
— — — **hacking**, short cough: Seneg.
— — — **hollow** and rough cough: Kreos.
— — — **morning**, on rising, cough from: Amm. bro.
— — — **paroxysmal** cough: Atropia.
— — —, **rising** into throat, excites cough: Asaf.
— — —, — — — causes difficult breathing and finally cough with expectoration: Asar.[7]
— — — — morning on rising a sensation of mucus in throat causes short cough: Am. bro.
— — — **rough** and hollow from: Kreos.
— — — — **short** cough: Senega.
— — — — short, hollow unceasing cough, excited by creeping, tick-

**THROAT**—(*Continued*).
ling, and by much mucus in throat; worse evening till midnight: Caust.[2]

— **mucus** in, short dry cough, with feeling as if mucus were hanging in throat and could not be loosened; an hour later the mucus loosens easily: Lauro.[1]

— — — **suffocative** cough with expectoration of much tough mucus, which accumulates in chest and throat, is difficult to loosen, causing almost strangulation and vomiting of food; worse night after going to bed and in morning while lying: Coc. c.[2]

— — — attacks of cough like whooping cough, excited by copious flat tasting, watery mucus, sometimes blood streaked, in chest and throat, which is difficult to dislodge and can be expectorated only in the morning: Euphr.[2]

— **pain** in, excites cough: Acon., Ang., Arg., Bry., Calad., Chin. s.,[1] Euphr., Grat., Hepar, Sars., Spong., Stann.—12.

— **pain** in, great dryness of, causing cough with aching pains in: Kalm.[7]

— — —, agg. when coughing: Arum. tr.

— — — — see also Soreness, Rawness, etc.

— **pain** in, on coughing: Arum tr.,[1] Caps., Carbo an.,[12] China,[12] Hepar, Iod., Kalm.,[7] Lach., Mag. s.,[12] Natr. m., *Nux v.*,[12] *Phos.*, Sil., Sulph.—5.

— — pain as if something would be torn loose in the throat, with violent cough, first dry, then saltish expectoration: Calc.[6]

**THROAT**, **pain**, cough in evening with pains in chest and throat, passing off when quiet: *Psor.*

— painful spot in, morning cough: Chin. s.

— **peculiar** sensation in excites cough, amel. by eating: Ammoniac.

— **plug**, cough as if from a plug in: *Calc.*,[1] *Lach.*, Spong.—5.

— — sensation as of a plug moving up and down in, cough: *Calc.*

— **pressing** upon, see Touching Throat.

— **pressure** in, with cough: Caps.[5]

— **pricking** in, with cough: Nitr. ac.[5]

— — — as from pin causing cough: Sil.[7]

— **prickling** in, exciting cough: Sol. t. ac.

— **raise**, she tries to raise something from throat, but the cough does not bring it up, on the contrary creates more of it: *Raph.*

— **rawness** in, excites cough; *Alum.*, Ambra, Bar., Cast., Coc. c., Laur., Nux v.—1.

— — — amel. by cough: Nicc.

— — — after dinner, cough from: Sil.

— — — dry cough from rawness in throat, during menses: Castor.

— — — dry cough, from continued tickling rawness in; Brom.

— — — hacking cough: Caust.,[7] Kali b., Kali iod., Stront.

— — —, in morning, cough: Ol an., Phos.

— — —, night, cough: Anac.

— — — paroxysmal cough: Coc. c.

— — and scraping in, on going to sleep, evening: Prun. sp.

— — in, short cough: *Acon.*, Ol. an.

— — tickling-rawness in, from, causing dry cough: Brom.

**THROAT**, **rawness** in, when coughing: *Arg.*, Carbo v., China, Kob., Natr. m., Phos.,[1] Rumex, Sep., Sil.,[1] Spong.,[1] Stront.[1]—12.

— — — pain in chest and bronchia, with rawness in throat when coughing: SPONG.

— — — chest and throat raw from dry spasmodic cough: Sil.[6]

— — — compare with Roughness in.

— **rise** in, after frequent short dry coughs, must swallow, something seems to rise up in throat; whining and moaning: *Cina.*[14]

— — — paroxysms of cough about midnight, something seems to rise in throat, as if it would choke her: *Cham.*

— **roughness** in, excites cough: *Alum.*,[1] Aur. m., Bar., Bry., Carbo an., *Carbo v.*, Caust., Colocn.,[1] Con., Dig.,[16] Graph., Kali. c.,[1] Kali iod., Kalm., Kreos., Laur., Mang., Natr. slfc.,[1] *Nux v.*,[16] Ol. an., Plumb.,[5] Puls., Rhod., Rhus,[5] Sabad., Sarsap., Senega,[5] Sulph.,[1] Stront.—12.

— **roughness** and crawling in, half involuntary cough: *Carbo v.*

— — in, **dry** cough: Kali iod.,[10] Natr. slfc., Verat. v.

— — — dry exhausting cough, morning and evening, with oppression of chest and roughness of throat: Rhod [7]

— — — dry cough from roughness or scratching in throat, especially in children, with lachrymation: Sabad.[7]

— — — dry cough forenoon: Sars.

— — — dry cough at night with roughness of throat and soreness of chest, has to sit up and hold chest with both hands: Natr. sul.[10]

— — — **hacking** cough: Ang., Rhus.

**THROAT**, **roughness** and scraping in, lying on right side, night cough: Sil.

— — — — —, short asthmatic tickle-cough, excited by a feeling of roughness and scraping in throat; worse forenoon and before midnight: Sabad.[2]

— — — — — spasmodic cough like whooping cough, excited by roughness and scraping in throat and in epigastrium; worse evening till midnight, warm food and in presence of a stranger: Bar.[2]

— — in, short cough: Verat. v.

— — in, when coughing: Ang., Arn.,[6] Carbo v., Caust., Dig., Gels.,[12] Hepar, Kali c., Kreos.,[12] Kob., Laur., Natr. sul., Nicc., Nitrum, Phos., Rhod., Sars., Senega, Sep., Spongia.—5.

— **scraping** in, excites cough: Æsc. h., Agn., Aloe, *Alumen*, Ambra,[5] *Amm. c.*, Arg. n., Bor., Bov., Cainca, Camph., Canc. f., Carbn. s., Chin. s., Coc. c., Croc.,[5] Cycl., Graph., Hal., Hepar,[6] Kalm., Laur., Mag. c., Pæonia, Petr., Phyt., Plat., Prun. sp., Puls., *Sabad.*, Senega, Tereb., Thuja, Upa.—1.

— — — **agg.** by coughing: Natr. c.

— — — agg. after eating, till 9 P. M.: Mezer.

— — — in open **air**, afternoon, cough: Phos.

— — — **dry** cough: Amm. c., *Bell.*, Bov., Carbo v., Graph., Sabad.

— — **dryness** in, causes tickling cough: Colch.

— — **evening**, hacking cough: Tereb.

— — **hacking** cough: Laur., Ol. an., Prun. sp., Sin. n.

— — — — evening: Tereb.

— — — **night**, cough: Con.

— — — paroxysmal cough: Coc. c.

**THROAT**, **scraping roughness** in, cough from: Kalm., Puls., Sabad.—5.
— — and rawness in, on going to sleep, in evening: Prun. sp.
— — and roughness in, on lying on right side, night: Sil.
— — — — short asthmatic tickle-cough, excited by a feeling of roughness and scraping in throat; worse forenoon and before midnight: Sabad.[2]
— — — — spasmodic cough like whooping cough, excited by roughness and scraping in throat and in epigastrium; worse evening till midnight, warm food and in presence of strangers: Bar.[2]
— **scraping** in, while coughing: Ambra, Bell., Bov.,[1] Bry., Calc. s.,[1] Croc., Dig., Hepar, Kreos.,[1] Laur., Mag. s.[1]—5.
— — — deep dry cough, with flow of water in, and subsequent scraping in throat: Ambr.[6]
— **scratching** in, excites cough: *Amm. c.*, Bart., Dig., Kreos., Mag. m., Nux m.,[7] Petro., Phos., Psor., Puls.—1 and 12.
— — —, belching causes scratching in throat, irritable larynx, and is followed by cough: Staph.[7]
— —, or crawling in upper part of wind-pipe: Nux m.[7]
— — in, **dry** cough: Acon., Bart., Psor.
— — — — — from scratching or roughness in throat, especially in children, with lachrymation: Sabad.[7]
— — — **dry** cough at night, coming deep from chest, caused by scratching in throat: Petr.[7]
— — — **extending** to sternum when coughing: Alum.
— — — **hacking** cough: Sil.
— — — **irritable** cough: *Phos.*

**THROAT**, **scratching** in, **on** coughing: Acon., Aloe.
— **sensation** as of a plug moving up and down in, cough from: *Calc.*
— **skin**, sensation as of a piece of loose skin hanging in throat, excites dry, hacking cough, frequent sneezing: Alum.[7]
— **smarting** in, cough from: Tarent.
— — —, during cough: Caps.,[5] Tarent.[1]
— **smothering** sensation in, cough from: Lact.[12]
— **soreness**, cough from: Kalm., Sulph.
— — agg. by coughing: Ran. sc.
— — while coughing: Ambra, Amm. c., *Arg.*, Caust., China, Flour. ac., Kobalt., *Lach.*, Lyc., Mag. s., Natr. m., Nitr. ac., Nux m., Phos., Ran. sc., Rumex, Sepia, Tarent (r).—1 and 12.
— — — throat sore, with tickling cough in evening, worse after going to bed: Calc. ph.[7]
— **spasm** of, so could only utter a hoarse croaking sound, alternated with a sonorous croupy barking cough: STANN.
— — —, with cough: Bell.[5]
— **spot**, dry spot in throat causes cough: *Cimic.*[7]
— — — — — larynx: CON.
— —, painful in, morning, cough: Chin. s.
— **sticking** in, causes irritation to cough: Phos.
— — griping in, causes irritation to cough: Mur. ac.
— — scraping cough: Lyc.
— **stiffness**, sensation of, in, with cough and suffocation felt in upper part of chest: Æsc. h.[14]
— **stinging**, severe, in uvula as if it would break, when coughs: Ham.

**THROAT**, **stitches** in, excite cough: Cham., *Cist.*, Sol. t. æ.,[1] Stann.—5.

— — — dry cough, not relieved by the cough: Caps.[6]

— — — — coryza with dry cough, headache and stitches in throat: Nitr. ac.[10]

— — —, when agitated or from mental excitement, excites cough: *Cist.*[8]

— — —, when coughing: Bry., Hepar, Kali c., Lyc., Nitr. ac., Phos., Sil.—1 and 5.

— **stretching** the, irritation to cough on: Lyc.

— **stuck**, sensation, while eating fruit, as if something stuck in throat causing cough: Arg.

— **sulphur** fumes, sensation as of, in, excites cough: Ars., Brom., Bry., Carbo v., China, Ign., Ipec., Kali chl., Lyc., Mosch., Par., Puls.—12.

— — constriction of, as if from sulphur fumes in pit of throat: Ign.

— **swelling**, a feeling of, in, excites cough: Ars.[5]

— — — danger of suffocation with swelling of throat at night, and cough: Ars.[6]

— — — with cough: Caps., Puls. —5.

— sweetish **taste** in, forenoon, while sitting, cough: Mag. c.

— **tearing** in, excites cough: Cist.

— **tickling** in, excites or agg. coughing: *Acon.*, Æsc. h.,[1] Æth.,[1] *Alum.*, *Ambra*, Am. bro.,[1] Amm. c., Amm. m., Anac., Ang., Ant. t., Arg. n.,[1] *Arn.*, Ars., Bapt.,[1] Bar., Bell., Bor., Bov.,[7] Brom.,[2] *Bry.*, Calc., Carbo v., Caust., Cent.,[1] *Cham.*, Cina, Cinnab., Colch., Coloc., *Con.*, *Dros.*, *Euphr.*, Ferr., Glon.,[1] Graph.,[6] Gymno., Hepar, Hippo.,[1] Hyos., Iod., Kali b.,[1] *Kali*

**THROAT**, **tickling** in—(*Cont'ed*). c., Lach.,[7] Lact., Laur., Lina.,[1] Lyc., Mag. c., Mag. m., Marum, Merc., Natr. c., *Natr. m.*, Natr. ph.,[1] Natr. s., Nicc., *Nux v.*, Olean. Ol. an., Phos.,[1] *Ph. ac.*, Physo.,[1] Phyt.,[1] *Prun. sp.*, Psor.,[1] Puls., Rhod., Rhus, *Rumex*, Sang.,[1] *Sars.*, Seneg., Sep., *Sil.*, Sol. n.,[1] Sol. t. æ.,[1] Spira.,[1] Squil., Stann., Staph., Sulph., Sum.,[1] Tabac., Thuja, Zinc., Zing.[1]—5 and 16.

— **tickling** in, cough at first caused by tickling in throat, gradually seeming to come from low down in throat, finally from chest; shook abdomen: *Sil.*

— — — tickling and tingling in throat causes cough, which is only relieved by discharging quantities of mucus from those parts: *Iod.*[14]

— — — **afternoon**, cough: Bov., Mag. c.

— — at **back** of, cough: Coca.

— — in, extending to **chest**: Sil.

— — —, **constant**, causes cough: Phos.

— — — — cough at night: Inu.

— — — — tickling in upper left side of throat, almost uninterrupted cough; worse talking, stooping, and toward evening and then suddenly ceasing: *Hepar.*

— — — **contractive** titillating sensation in throat from larynx to lowest bronchial tubes: *Ipec.*[6]

— — — **convulsive** cough, night: Merc. c.

— — crawling in, causes dry cough: Calc. ph.

— — in, croupy, rough, barking or whistling cough, excited by tickling in throat and, as if, by vapor of sulphur: Brom.[2]

— — in **deep** cough: Am. bro.

**THROAT**, **tickling** in, before **dinner**, cough: Arg. n.

— — — **dry** cough: Bor., Ferr., Indigo, Plan., *Phos.*, Sepia.

— — — dry cough from tickling crawling in throat: Calc. ph.

— — —, dry cough day or night, from: Amm. m.[7]

— — — dry incessant, gagging cough, from; worse on falling asleep and during day: *Lach.*[7]

— — — dry cough, evening: Amm. m., Arg. n., Gymno., Sulph.

— — — dry cough forenoon: *Amm. m.*

— — — — — frequent dry cough from tickling in throat, accompanied by constriction of chest; and dryness in throat; the cough is forcible, concussing abdomen and head: Lact.[2]

— — — dry cough night: Crot. c.

— — — **evening**, cough from: China, Zing.

— — — — 5 P. M.: Bov.

— — — —, on lying down: Nicc.

— — — — with cough and headache: Sang.[10]

— — — — **dry** cough: Amm. m., Arg. n., Gymno., Sulph.

— — — **dry** hollow cough, with soreness in chest, caused by tickling and mucus in throat, with expectoration, only at night, of acrid tasting mucus, which has to be swallowed: Caust.[10]

— — — — hacking cough: Amm. m., *Sang.*

— — — — hard cough: Gymno.

— — — — exhausting cough before retiring: Arg. n.

— — — **exhausting** cough, evening, before retiring: Arg. n.

— — — **fatiguing** and frequent cough from tickling in throat, evening after lying down: Sang.[2]

**THROAT**, **tickling** in, **feather**, forenoon, cough from tickling as if by a: Glon.

— — — **forenoon**, dry or hacking cough: *Amm. m.*

— — — **grease**, titillation in throat with sensation as if throat were irritated by the smoke of rancid grease, causing cough: Hepar.[6]

— — — **hacking** cough: Colocn., Mur. ac., *Phos.*, Plan.

— — — — — evening: Amm. m., *Sang.*

— — — — — — lying down: SANG.

— — — — — morning: Sum.

— — — **hard** cough, evening: Gymno.

— — — causes **irritation** to cough: Aspar., Natr. c.

— — — — — — — 9 P. M.: Dios.

— — **low** in, deep cough: Dios.

— — — — forenoon: Dios.

— — — — hacking cough: Dios., Tarax.

— — in, **morning**, 8–9 A. M., cough: Sil.

— — — — 9 A. M.: Sepia, Tarent.

— — — —, in bed: Coc. c.

— — — **morning**, dry cough: Amm. m.,[10] Ox. ac.

— — — — at 3 A. M., dry cough from tickling in throat, as of dust: *Amm. c.*[10]

— — — — hacking cough: Sum.

— — — — paroxysmal cough, in bed: Coc. c.

— — — — after rising: Alum.

— — — **night**, dry cough: Crot. c.

— — — **paroxysmal** cough: Caust.

— — — — — morning, in bed: Coc. c.

— — — **racking** cough, caused by titillation in throat, feels as if chest would fly to pieces: Lact.[6]

**THROAT**, **tickling** in, **rawness** causes dry cough: Brom.
— — in **short** cough: Colocn., Iod., Mag. c.
— — —, worse at **sides** of, cause sudden cough, evening: Amm. bro.
— — — while **sitting**, cough: Mur. ac.
— — — — — dry cough: Con.
— — — **spasmodic** cough, attacks of spasmodic cough, excited by tickling in throat; evening until after midnight: Magn. m.[2]
— — — — asthmatic, hacking, spasmodic cough, in short but frequently recurring paroxysms, excited by tickling in throat and larynx; worse at night, especially after midnight: Kali c.[2]
— — — — hollow, hacking, spasmodic, tickling cough, excited particularly by a tickling itching in chest or throat; worse evening or night: Phos.
— — — — hollow or whistling, spasmodic cough, excited by roughness, scratching, and tickling in chest and throat; worse morning and evening: Kreos.[2]
— — — — powerful, spasmodic, nocturnal paroxysms of whooping cough, excited by itching and tickling in chest and throat, or as if by a dry spot in larynx; worse at night, and when lying down: Con.[2]
— — — — spasmodic cough at night, occurring in paroxysms every quarter of an hour, each paroxysm consisting of but few coughs, with a rough, hollow, barking tone, excited by tickling in throat, as if from down, or as if from constriction of larynx; worse evening and night: Bell.[2]

**THROAT**, **tickling** in, violent spasmodic cough, short consecutive coughs, caused by a tickling sensation in throat, as if some mucus were lodged in it: Hyos.[10]
— — — — spasmodic whooping, excited by tickling in throat and epigastrium; worse evening after lying down, less in morning: Natr. m.[2]
— — — **sudden** cough from tickling in throat; worse at sides, evening: Amm. bro.
— — — **suffocative** cough from tickling in throat: Hepar.
— — at **top** of, dry cough, noon: Naja.
— — in upper part of, cough: *Hepar.*
— — beneath thyroid cartilages, dry cough: *Squil.*
— — in region of thyroid cartilage, short cough: *Puls.*
— **tingling** in, excites cough: *Iod.*[5]
— **titillation**, desire to clear the throat, with rough feeling in throat, as if mucus were adhering to it, with titillation inducing cough: Lyc.[6]
— titillation with cough as from fumes of sulphur in throat, with gray salt expectoration: Lyc.[6]
— **torn**, pain on coughing, as if throat would be torn: Calc., Phos.—6.
— — — as if something would be torn loose in throat, with violent cough, first dry, then with saltish expectoration: Calc.[6]
— **touching**, agg. or excites coughing: *Bell.*, China, LACH., *Rumex*, Staph.
— **touching** excites dry or hacking cough; also occurring in the morning after sleep: *Lach.*
— **ulcer** in, cough from: Lach.

**THROAT**, ulcerative pain in, from cough: Caps.[5]
— **upper part**, choking contraction in, causes cough: Cocc.
— — tickling in upper part of throat causes cough: Hepar.
— — tickling at top of, causes dry cough, noon: Naja.
— **weight**, feeling of oppression (weight) from throat to chest preceding the raising of mucus, cough with: Iod.[6]
— **wheezing**, deep, rough, fatiguing cough, with rough voice, wheezing in throat; raising of salt-sweetish mucus: Mag. m.[6]
— **wide** enough, when coughing throat does not seem: Cocc.[6]
— **worm**, sensation as if a, crawled up from pit of stomach in throat, causing the cough; cough worse at night, with shooting in scrobiculum: Zinc.[8]

**THROAT-PIT**, affected chiefly by the cough: Eug. jamb.[14]
— **burning** in, excites cough: Ars.[2]
— **commencing** in throat-pit, a hacking cough, with a cool, saltish liquid being lodged in the lower part of the throat: Cann. s.
— **constriction**, as from sulphur fumes in throat, cough: Ign.
— **crawling** in, excites cough: Mag. m.
— **crawling** irritation near suprasternal fossa, before midnight, causing cough; amel. by swallowing mucus: Apis.
— — and tickling in, dry cough: *Sang.*
— — paroxysmal cough, before midnight, causing pain in head, from crawling in windpipe near supra-sternal fossa: Apis.
— **down**, spasmodic tickle cough, excited, as it were, by down in larynx, supra-sternal fossa, and in whole chest as far as epigastrium; worse morning and evening: Ph. ac.[2]
— **dust**, dry cough as from dust in pit of throat, agg. evening and by coughing: *Ign.*
— **hacking** cough, arises from throat-pit, with a cool salty fluid deep in throat, posteriorly: Cann. s.[7]
— **irritation** in, excites cough: Apis,[1] Bell., *Cham.*, Croc., IGN., Iod., Lac. can., Ph. ac., Rhus r., Rumex, Sil.—2 and 12.
— — hollow, spasmodic cough, excited, in evening, by an irritation in supra-sternal, as if from vapor of sulphur, or from down, and in morning by a tickling just above epigastrium: Ignat.[2]
— **itching** tickling in, excites deep cough: Sil.
— **mucus**, cough which sounds as if a little cupful of mucus were in throat-pit: *Ant. t.*
— **sulphur** fumes, constriction as from, in throat-pit, cough: Ign.
— **tickling** in, excites cough: Bell., Cann. s.,[1] *Cham.*, Cinnab., Cocc., Coloc.,[12] Crot. h.,[1] Ign., Lac. can.,[12] Lach., Ph. ac., Puls., Rhus r., *Rumex*, *Sang.*, *Sil.*, Squil., Tarax.—5.
— **tickling** itching in, excites deep cough: Sil.
— — in, dry cough: Inu., Sang.[7]
— — and crawling in, dry cough: SANG.
— — dry, incessant, fatiguing cough, caused by tickling in throat-pit, extending behind sternum and to stomach (worse when walking[10]) (or as if from an awn of barley swaying to and fro in one bronchi): *Rumex.*[7]

**THROAT-PIT, tickling** dry spasmodic cough, assimilating the early stage of whooping cough; at first dry (about two weeks later, loose); came on in paroxysms, preceded by a tickling sensation in throat-pit (and attended by pains in head and chest); worst paroxysm came on a few minntes after lying down at night, usually about 11 P. M.; less severe paroxysm occurred in morning on waking: *Rumex.*[1]

— — dry cough, with tickling in throat-pit, and a crawling sensation down beneath sternum (cough becoming very severe, causing pain beneath sternum): SANG.[1]

— — shattering cough from: Sil.

— — spasmodic cough excited by intolerable tickling in larynx and in supra-sternal fossa; worse in morning and from vexation: Iod.[2]

— — suffocating, hollow cough, resembling whooping cough, provoked by tickling in chest, larynx, throat, and supra-sternal fossa; worse at night and from anger: Cham.[2]

— compare Throat and Trachea.

**TICKLING** cough: Alum.,[5] *Ambra*, Anac., Ang., Ant. t., *Arn.*, Asaf.,[5] Bell., Bov., Brom.,[1] *Bry.*, Calc., Calc. ph.,[1] Canth.,[1] *Carbo an.*,[1] Carbo v., *Caust.*,[1] Cham., China,[5] *Coca*,[1] Coc. c.,[1] Colch., Coloc., *Con.*, *Dros.*, Erio.,[1] *Euphr.*, Ferr., *Hyos.*,[1] Ipec., Kali b., *Kali c.*, Kali hypermang., *Lach.*,[1] Laur., Linu., *Lyc.*, Mag. c., Mag. m., Marum, Merc., Naja, Natr. c., *Natr. m.*, Natr. ph.,[1] Nitr. ac., Nitr. d. s.,[1] *Nux v.*, Olean., Ol. an., Ol. jec.,[1] Petro., *Phos.*, Ph.

**TICKLING** cough—(*Continued*). ac., Rhus, Rumex, Sabad., *Sabina*, Sars., *Senega*, *Sepia*, Sil., Spong., *Squil.*,[5] Stann., *Tabac.*, Thuja, Zinc.[1]—12.

— — **agg.** in open air: *Lach.*

— — — evening in bed: Calc. ph.

— — — — on going to sleep: Lyc.

— — — morning: Lyc., Thuja.

— — — night: Rhus, Zinc.

— — open **air**, agg. in: *Lach.*

— — — teasing cough and frothy mucus expectoration when in open air: Linum.

— — **amel.** 6 P. M. by expectoration of mucus: Sulph.

— — **blood**, tickling cough with taste of blood, on waking: Ham.[7]

— — **day**: Coloc., Lyc.

— — **evening**: Alumen, Calc. ph., China, Coloc., Natr. m.

— — — 3 P. M.: Hepar.

— — — 6 P. M., amel. by expectoration of mucus: Sulph.

— — — agg. in: Merc., *Rhus.*—12.

— — — agg. evening in bed: Calc. ph.

— — — — on going to sleep: Lyc.

— — **expectoration** of mucus amel., 6 P. M.: Sulph.

— **harassing** cough: *Squil.*[5]

— cough, **larynx**, from sensation of sulphur fumes in: LYC.

— — **morning**: Coloc.,[6] Natr. m.[1]

— — — 3 A. M.: Cainca.

— — — agg. in: Lyc.,[1] Thuja.[12]

— — — after rising: Alumen.

— — — after waking: Carbo v.

— — **night**: Arg. n., *Coloc.*, Natr. m.

— — — agg. at: Rhus,[12] Zinc.[1]

— — — night and day: Natr. m.[12]

— — — dry hard tickling cough, worse evening till midnight, with stiffness in back and limbs: Rhus.[7]

— — **palate**, from tickling in soft palate: Fran.

**TICKLING** cough, **pharynx**, from sensation, as if acid or acrid liquid flowed from choanæ to pharynx: Kali b.
— — from **smoking**: Atropia, Coloc.[6]
— — **sneezing**, constantly, with sharp pains in centre of temples, tickling cough, light, mushy stools: Iris v.[7]
— — **sternum**, from irritation behind; stitches in left lung: Rumex[10].
— — on **talking**: Alumen, Atro.
— **teasing** cough and frothy mucus expectoration when in open air: Linum.
— cough, **throat**, from great dryness in: SANG.
— — — from sensation of scraping dryness in: Colch.
— — — from sensation, as if a feather in: *Calc.*
— — — sore throat, with tickling cough in evening; worse after going to bed: Calc. ph.[7]
— — **trachea**, from mucus in; at night after lying down inability to sleep; hoarseness: Arum tr.[7]
— — — from soreness low in: *Stann.*
— — **waking**, in morning after: Carbo v.

**TIGHT** cough: Calc. s.
— — day: Natr. ars.
— — evening: Calc. s.
— — on deep inspiration: Ziz.
— — from dryness of larynx: Ziz.

**TOBACCO**, see Smoking.

**TONELESS** cough: Calad., Card. b.

**TONGUE**, burning on, causes irritation to cough: Acon. f.
— rawness and scraping on back of, excite cough: Calc. ac.
— sensation, as if hair on, causes cough: Sil.[12]

**TONSILS**, dryness and sticking in, cause cough: Stann.
— hacking cough on touching swollen: Phos.
— pain when coughing: Amm. bro.
— sudden cough from tickling below: Amm. bro.
— tickling below, afternoon and evening: Amm. bro.

**TOOTHACHE** when coughing: Lyc.,[1] Sepia.[11]

**TORMENTING** cough: Alum., Amm. c., Anac., Ang., Ars., Arum tr., Asaf., Bar., Bell., Bor., Brom., *Calc.*, Cann. s., Carbo an., Caust., Chelid., China, Cina, *Cocc.*, Con., Coral., *Croc.*, Cupr., Daph., Dros., Dulc., Hepar, Hydro. ac., Ipec., Kali c.,[7] Lach., Lact., Led., Lobel., Merc., Merc. c., *Mezer.*, Mur. ac., Natr. c., Natr. m., Nitr. ac., *Nitrum*, *Nux v.*, Op., *Phosp.*, Rhod., Selen., Sep., Spig., *Squil.*, Stann., Sulph., Verat.—12.
— dry cough: Nitrum.[5]
— cough, gets nothing up; sometimes feels as if a tough membrane were moved about, but would not loosen: Kali c.[7]

**TRACHEA**, also compare with symptoms given under Throat.
— **breath**, sudden stoppage of, in upper part of trachea, excites violent cough: Ign.[6]
— — on breathing irritation to cough, as from mucus in trachea (felt more while sitting bent over than when walking): STANN.
— **burning** in, excites cough: Acon., Ars.—12.
— burning tickling in upper part of trachea, cause cough: Euphr.
— **coldness**, cough with, in: Brom., Sulph.—12.
— **constriction** of,causes irritation to cough: *Cocc.*,[6] Mosch., Stann.

**TRACHEA**, **constriction**, spasmodic cough, excited by a feeling like constriction of chest and trachea, as if from vapor of sulphur: Mosch.[2]

— **contractive** sensation in trachea as if irritated by smoke, inducing almost constant cough: Cocc.[6]

— **crampy** pain in trachea and bronchi preceding the cough: Ind.[6]

— **crawling** in, cough from: Colch.

— — — upper part of, cough from: Carbo v., Prun. sp.

— **creeping** in, excites cough: Anac., Arn., Carbo v., Caust., Kreos., Rhus.—12.

— **cutting** coolness in, before the cough: Camph.[6]

— — — short hacking cough, as if caused by a cutting coolness in trachea: Camph.[6]

— — stinging in, with mucus in chest, cause cough: Arg.[10]

— **deep** cough from, morning: Angust.

— **dryness** in, cough from: Carbo an., Laur., Puls., Stann.—2.

— — suffocative cough from dryness and scraping in trachea, in open air: Cycl.

— — suffocative, hoarse cough, excited by rawness and dryness in larynx and trachea, with gray, greenish sometimes purulent, day expectoration; worse evening and night: Carbo an.[2]

— — a tickling cough, deep, hollow, concussive, and periodically recurring, in paroxysms consisting of three successive coughs, excited by mucus in chest and (in evening) by stitches and dryness in trachea: Stann.[2]

— **dust**, sensation of, in trachea and behind sternum, not relieved by coughing: Chelid.[2]

**TRACHEA**, **feather** or awn of barley in one of the bronchia, and swaying to and fro, with perspiration, sensation as of a: Rumex.[2]

— **foreign** body, tracheal cough as from, with cramp-like feeling in larynx: Tereb.

— **hair**, sensation of a, in trachea, excites cough: Sil.

— **heat** in, frequent paroxysms of dry, violent, hollow, or short exhausting cough, excited by severe tickling in larynx, which brings tears to the eyes; by heat in trachea, and by a sensation of dust in trachea, throat, and behind sternum, which is not relieved by coughing: *Chelid.*[2]

— **irritation** in, excites or agg. cough: Acon., Agar., Alum., Ang., Ant. t., Arg., *Arn.*, Ars., Asaf., Bar., Bell., Bov., *Bry.*, Calc., Cann. s., Carbo an., Carbo v., Caust., Cham., China, Cina, Cocc., Colch., Coloc., Con., Croc., Dig., Euph., Ferr., Graph., Grat., Hepar, Hyos., Ign., Iod., Ipec., Kali b., Kali c., Kali iod.,[12] Laur., Led., Lyc., Mag. c., Marum, Merc. sol., Mur. ac., Nicc., Nitr. ac., Nitrum, Nux m., Nux v., Petro., Phos., Prun. sp., Psor., Puls., Rhod., Rhus, Sabina, Senega, Sepia, Sil., Spig., Squil., Stann., Staph., Stront., Sulph., Thuja, Verat., Zinc.—1 and 5.

— **irritation** in, as from dust, after dinner, when washing, dry cough: Ferr. mag.

— — — **dry** cough, forenoon: Coc. c.

— — — — — as from dust in, after dinner, when washing: Ferr. mag.

— — — **evening**, cough: Petrol.

— — — **forenoon**, cough: Sulph.

— — — — dry cough: Coc. c.

**TRACHEA**, **irritation** in, **forenoon** on waking, cough: Dros., Natr. m.

— — — — after waking, cough: Rhus.

— — — **hacking** cough: Trif. p.

— — — **low** down in trachea, cough from: Spig.

— — — — irritation to cough, low in trachea, sore sensation in chest and trachea after every cough: STANN.

— — — **morning**, in bed, cough: Sulph.

— — — — short cough: Coc. c.

— — **prickling** irritation, violent paroxysm of cough, or frequent cough excited by prickling irritation, which begins in larynx and extends thence down into trachea, followed by dryness of mouth and larynx: Hydro. ac.[2]

— — in, **short** cough from, morning: Coc. c.

— — short cough from, in upper trachea: Euph.

— — in, **tracheal** cough, as from: Croc.

— — — **upper** trachea, excites short cough: Euph.

— — — **warm** room, on entering, from open air, cough: *Bry.*

— — — **washing**, dry cough from irritation in trachea, as from dust, after dinner, when washing: Ferr. mag.

— — — **whooping** cough excited by irritation in trachea as if from sulphur vapor; worse evening, from 4 to 8 o'clock, and again about midnight: Lyc.[2]

— **itching** in, excites cough: Cham.,[6] Con., Puls.—2.

— — — cough caused by itching in trachea and extending from pit of stomach to epiglottis: *Puls.*

— — — shattering, spasmodic

**TRACHEA**, **itching** in,—(*Cont'd*).

whooping cough, often in paroxysms of two coughs each, excited by an itching scratching, with a feeling of dryness, and, as it were, of vapor of sulphur in trachea and chest; worse evening till midnight: Puls.[2]

— — tickling in upper part of trachea, causing dry cough: Cham.

— **mucus** in, excites cough: *Arum tr.*,[7] Caust., Cham.,[5] Cina, Crot. t.,[1] Cupr., Dulc., Euphr., Gels.,[1] Hyos., Nux v.,[5] Squil.—12.

— — **adherent** high up in, cough from: *Nux v.*

— — accumulation of mucus in trachea is easily thrown off by coughing; afterward, soreness or stitches in chest: Stann.[7]

— — in, on **breathing** irritation to cough, as from mucus in trachea (felt more while sitting bent over than when walking): STANN.

— — — **forenoon**, easily expelled by a forcible cough, with great weakness in chest, as if eviscerated, and with weakness of body and limbs: STANN.

— — — **hacking** cough from, while talking: Gels.

— — — **laughing** causes mucus in trachea and cough: Arg.[10]

— — — mucus gets in trachea when going up-stairs or stooping, which is expelled by a single cough: Arg.[6]

— — — **rattling** of mucus high up in, cough from: Cham.

— — — **tickling** cough from mucus in trachea; at night after lying down inability to sleep; hoarseness: Arum tr.[7]

— — — violent, short paroxysms of spasmodic cough, excited by mucus in trachea, and by creeping

**TRACHEA**, **mucus** in,—(*Cont'd*).
sensation in chest; worse after midnight and in morning: Squil.[2]

— — — **whooping** cough, excited by excessive secretion of mucus in larynx and trachea: Dulc.[7]

— **noise** in, after coughing: Lach.[6]

— **pain** in, excites cough: Acon., Ang., Arg., Bry., Calad., Euph., Grat., Hepar, Ipec., Sars., Spong., Stann.—12.

— — clawing pain in trachea, gradually descending into bronchi causing cough, with expectoration of small balls of tenacious mucus: Indigo.[5]

— — piercing pain in, excites cough: Acon., Stann.—2.

— — in, from coughing: Camph., China, Ign., Laur., Nux v., Spongia.—5.

— **pressure** of fingers on, cough from: Hydras., *Lach.*, Rumex.

— **pricking** in, excites cough: Hydro. ac.[5]

— **rawness** in, excites hacking cough: Laur.

— — dry cough with raw feeling in entire trachea: Grat.[6]

— — suffocating, hoarse cough, excited by rawness and dryness in larynx and in trachea, with day expectoration of gray, greenish matter; worse evening and night: Carbo an.[2]

— **roughness** in, cough from: Bar., Carbo an., Dig., Kreos., Sabad.—2.

— — hollow, deep, spasmodic cough, excited by roughness and scratching in roof of mouth and in trachea: Digit.[2]

— **scraping** in, excites cough: Thuja.

— — — before and after the cough: Laur.[6]

**TRACHEA**, **scraping** in, dry cough from: Bry.

— — and dryness in, in open air, causes suffocative cough: Cycl.

— — in, short cough from: Sabad.

— **scratching** in, excites cough: Dig., Kreos., Puls.—2.

— — — dry cough from: Agar. m.

— **sensation** of something in trachea causes short or hacking cough: Sin. n.

— sensation as if something were adhering in trachea, which cannot be detached by coughing: Hyos.[6]

— **smoke**, sensation in trachea as if full of smoke, when entering warm room from cold, causes cough; feels as if he could not inhale sufficient air: Bry.[6]

— — contractive sensation in trachea, as if irritated by smoke, inducing almost constant cough: Cocc.[6]

— **soreness** in, after coughing: *Bry.*

— — — causing dry cough: Upa.

— — — extending to middle of sternum, after coughing: Osm.

— — — **during** cough: Caust., Iris v., Nux v., *Stann.*

— — —, and chest from scraping cough, excited by irritation low down in trachea; greenish expectoration of an offensive, sweetish taste, voice hoarse; worse evening just before lying down: STANN.

— — — dry cough, in from five to six paroxysms, with sore sensation in a streak down along the trachea, where it pains at every cough, almost preventing breathing: CAUST.

— — — a spot from which mucus is loosened by the cough, amel. on rising from bed: Nux v.

**TRACHEA**, **soreness** in, **tickling** cough from soreness low in: *Stann.*

— **stinging** cutting in, causes cough, with mucus in chest: Arg.[10]

— **stitches** in, causing cough: Arg., Lach.

— — and dryness in, evening, cause cough: Stann.

— — dull stitch in, from below upward, causing cough with watery expectoration: Arg.[6]

— — violent cough and painful stitches in trachea from slightest exertion: Manc.[7]

— **sulphur**, dry cough from irritation in trachea as from sulphur fumes, with pain in stomach: Lyc.[7]

— **tickling** in, compare with irritation in, also with irritation and tickling as given under Throat.

— **tickling** in, excites or agg. cough: *Acon.*, Amm. m., Anac.,[7] Ant. t., *Arn.*, Ars., Arum tr.,[7] Asaf., Bar., Bell., Brom., Bry., Calc., Caust., Cham., China, Coloc., Con., Cop.,[1] Digit.,[1] Euphr., Ferr.,[5] Gymno, Hyos., Kali c., Lach., Lact., Laur.,[1] Mag. c., Mag. m., Marum,[5] Mezer., Natr. m., Nicc., Nitr. ac., Nux v., Ol. an.,[1] Ox. ac.,[1] Phos., Ph. ac., Psor., *Puls.*,[1] Rhus, Sang., *Senega*, *Sepia*, Sil., Stann.,[1] Staph., Verat, Verb., Zinc.—2 and 12.

— **tickling** at bifurcation of, cough from: *Kali b.*

— — burning tickling in upper part of trachea, cough from: Euphr.

— — in, **dry** cough from: Aur. mur., Carb. ac., Petrol., Psor., Rhod.

— — — **dry** cough, evening: *Still.*

— — — — — from tickling itching in upper trachea: Cham.

**TRACHEA**, **tickling** in, **dry** cough low in trachea, morning: *Arn.*

— — — — — midnight: Phosph.

— — — — — morning, tickling low in trachea: *Arn.*

— — — severe, dry, racking cough caused by tickling in right side of trachea, below larynx: Sticta.[7]

— — — dry scraping cough from, with sleeplessness from midnight till 4 A. M.: Nicc.[10]

— — — dry cough, upper trachea, tickling in, which is agg. by the cough: Marum.[7]

— — — — — on entering warm room, in evening: Com.

— — — **eating** relieves; tobacco smoke agg.: Euphr.[7]

— — — **evening**, cough in, from: Bov.

— — — — dry cough: *Still.*

— — — — dry cough, on entering warm room, from: Com.

— — — — irritable cough, after lying down: Marum.

— — — on **expiration** cough from: Nux v.

— — — as from **feather** in trachea, cough: Prun. sp.

— — — **hacking** cough: Carb. ac.,[1] Psor.[7]

— — — — — morning after rising, from tickling low in: *Arn.*

— — — — — on entering warm room, evening: Com.

— — — **hard** cough: *Psor.*

— — — hoarse, ringing cough, excited by tickling in trachea, with asthmatic feeling therein; spasmodic contraction of thorax and accumulation of stringy mucus: Asaf.[7]

— — — on **inspiration**, cough: *Brom.*, Prun. sp.

— — — **irritable** cough from, after lying down, evening: Marum.

**TRACHEA**, **tickling** in, **low** in, dry cough from tickling low in trachea: *Arn.*, Cina,[6] *Verat.*

— — — **midnight**, dry cough: Phosp.

— — — **morning,** cough: Bov., Sepia.

— — — — after rising, cough from tickling low in: *Arn.*

— **tickling** in, short cough from tickling in region of thyroid cartilage: *Puls.*

— — — attacks of hollow spasmodic cough, excited by tickling in larynx and trachea: Staph.[2]

— — — spasmodic cough, which stops breathing, produced by tickling in trachea; cough is violent, dry, hollow, and is agg. by stooping forward: Spig.

— — — exhausting, spasmodic cough, excited by tickling in larynx and in trachea as far down as middle of chest; worse afternoon and evening until midnight: Zinc.[2]

— — — spasmodic cough excited by tickling in trachea; worse evening until midnight, during this period sputa cannot be dislodged, but in day, during motion, it becomes loosened: Ferr.[2]

— — — spasmodic cough, excited by tickling in larynx, trachea, and in thyroid region; yellow tenacious day expectoration; worse evening until beyond midnight: Mag. c.[2]

— — — short, spasmodic cough, in brief but frequently repeated paroxysms, excited by tickling as if from feathers or down in throat and trachea; copious yellow or grayish mucous expectoration with sour taste; worse evening and night: Calc.[2]

**TRACHEA**, **tickling** in, shattering spasmodic whooping cough, with frequent rapidly succeeding coughs, excited by tickling as if from mucus firmly seated in trachea; worse at night, especially after midnight: Hyos.[2]

— — — spasmodic, shattering cough, excited by tickling creeping in larynx, trachea, and chest; worse evening until midnight: Rhus.[2]

— — — short, wheezing, or hard cough, excited by insupportable tickling in larynx, or by tickling at bifurcation of trachea, or by oppression at epigastrium, or by accumulation of mucus in chest; sometimes dry, generally of tough mucus, yellow, greenish or black; worse on awaking: Kali b.[2]

— — — whistling, spasmodic cough, in single paroxysms, frequently returning, excited by tickling in larynx and trachea, as if they were dry, without sputa; worse toward evening: Lauro.[2]

— — — **upper** part of trachea, causes short dry cough, which is worse from coughing: *Marum.*[7]

— — — **warm** room, on entering, evening, tickling in trachea, dry cough from: Com.

— — — — — sensation of vapor in trachea on going into warm room from open air, causing cough: Bry.

— **tightness**, dry cough with painful tightness on left side of trachea and œsophagus, extending obliquely through right breast, and causing a concussion in this part: Cimex.[6]

— **tingling** in, evening, cough from: Stann.

**TRACHEA, upper part,** crawling in, cough from: Carbo v., Prun. sp.
— — **burning** tickling in, cough from: Euphr.
— — **itching** tickling in, dry cough from: Cham.
— — **tickling** in, dry cough: Marum.
— — — — irritable cough: Marum.
— — — — short cough from irritation in: Euph.
— — — — tracheal cough: *Bry.*
**TRACHEAL** cough: Carbon. s., Hyosn.
— — as from foreign body: Tereb.
— — — — irritation: Croc.
— — tickling low down, morning after rising: *Arn.*
— — — in upper trachea: *Bry.*
**TREMOR**, cough with: Phos.[12]
**TROUBLESOME** dry cough: Ign.[5]
**TRUMPET**-toned cough: Verb.[5]
**TURNING** from left to right side, amel. cough: Thuja.[5]
**ULCERS**, cough excites pain in: Con.[6]
— on legs with cough: Selen.[6]
**UMBILICUS**, colic as if, would be torn out, with continual cough; heat in face and sweat on forehead; after walking in open air and when lying, morning and evening: *Ipec.*[6]
— pain at, agg. by coughing and deep inspiration: Lyc.
— pressure in, when coughing: Ambra.
— stitches from umbilicus to genitals, on coughing: Sepia.
— compare with Abdomen.
**UNCOVERING** (undressing, etc.), agg. or excites cough: Chel.,[1] Hepar,[7] Kali b., Nux v., Rhus, Sil.—5.
**UNCOVERING** feet or head: SIL.[5]
— hands: *Hepar,*[7] *Rhus.*[5]
**UNINTERMITTING** cough: *Cupr.*
**UNINTERRUPTED** cough, cannot speak a word, discharge of bloody mucus from nose, after sea wind: Cupr.[7]
— see Constant Cough.
**URGENT** cough: Con.
**URINE**, involuntary discharge of, when coughing: Alum., *Ant. cr.*, Apis, Bell., Bry., *Caps.*, Carbo an., CAUST., Colch., Dulch., Hyos., Ign., *Kreos.*, Laur., Mag. c., *Natr. m.*, Nitr. ac., Phos., Ph. ac., Puls., Rhod.,[7] Rhus, Spong., *Sepia,*[12] SQUIL., Staph., Sulph., Tarent.,[1] *Verat.*, Zinc.—2 and 5.
**UTERUS**, pain in, on coughing: Bell.[5]
See under Abdomen.
**UVULA, elongated,** cough from: Alum., Bapt., Merc.-i-rub.—7.
— severe stinging in, as if it would break, on coughing: Ham.[7]
**VAULTS**, air of, agg. cough: Ant. t., *Sepia,* Stram.—5.
— cough from living in cool, dark places: Nux m.[7]
**VERTIGO** on coughing: Acon., Ant. t., Calc., Coff., Cupr., Kali b., Led., Naja,[1] Nux v.—5.
— oppression of head and chest, with vertigo: *Brom.*[6]
**VEXATION**, cough after: Acon., Ars., Bry., CHAM., China, IGN., *Natr. m.*,[8] Nux v., Sep.,[8] STAPH., Verat.—5.
— with chagrin: Ign.[5]
— with fright: Acon., Ign.[5]
— with indignation: *Staph.*[5]
— after slight vexation, she cried the whole night and coughed,

**VEXATION**—(*Continued*).
with ineffectual retching: Natr. m.[6]
— wheezing and coughing after: Natr. m.[8]
**VINEGAR**, cough after using: Ant. cr., Sepia, Sulph.—5.
**VIOLENT** cough: Alum.,[2] Ambra, Amm. m., Anac., Ang., Ant. t., Bell., Bry., Calc., Carbo an.,[6] Carbo v.,[6] Chelid., China, Cina, Clem., Con., Coral., Croc., Cupr., Dros., Eupat. per., Hepar, Hydro. ac.,[2] Hyos., Ign., Indigo, Ipec., Kali b., Kali c., Kali chl., Lach., Lact.,[2] Led., Lith., Lobel.,[2] Merc., Mezer., Mur. ac., *Natr. c.*, Nicc., Nitr. ac., Nux v., Olean., Par., Petr., Phosp., Plat., Puls., Rhus, Rumex, Ruta, Sabad., Senega, Sil., Spig., Spong., Squil., Stann., Staph., Stront., Verat.—5.
— — **agg.** afternoon, evening, and on entering warm room: *Natr. c.*
— — — laughing, talking, and yawning: Mur. ac.
— — **chest**, from constriction across: Ipec.
— — — from dryness of: Kali chlo.
— **continuous** cough: IPEC.[5]
— — — till relieved by vomiting: MEZER.[5]
— cough, convulsive and violent, accompanied by eructations and hoarseness: AMBRA.
— — after **dinner**: Mur. ac.
— **dry** cough, beginning at 3 A. M.: KALI C.[5]
— **dry** cough, on entering warm room from cold air: Natr. c.[10]
— **dry** racking cough, from tickling in larynx, lasting ten minutes; relieved by a glass of water; it, however, returns in fifteen minutes with so great violence that tears come into eyes, again relieved by water: Opium.

**VIOLENT cough**—(*Continued*).
— — in exhausting paroxysms: HYOS.[5]
— — proceeding as if from small spot in epigastrium; painful to the touch: Kali b.[6]
— — **evening**, before going to bed, continued violent cough: Con.[6]
— — **evening** after lying down: *Kali c.*
— — **evening**, in quick shocks, while lying, must rise: Lith. c.[7]
— — **exertion**, violent cough and painful stitches in trachea from slightest exertion: Manc.[7]
— — **hypochondrium**, cough with yellow purulent expectoration and stitches in left: Carbo v.
— — **meal**, after every: *China.*[6]
— — **morning**, early, in bed, with expectoration of quantity of mucus: Bry.[6]
— — — in morning he is obliged to cough so violently in order to detach the mucus that his eyes fill with tears: Cina.[6]
— — at **night**, from tickling in larynx: Cycl.
— — — nightly very violent cough, succeeded by hoarseness, with chilliness from morning till evening: Cupr.[6]
— — — violent, dry, nightly cough, with tearing pains in head and stitches in chest, followed by palpitation of heart: Cupr. acet.[6]
— — **noon**: Bell.[7]
— rattling cough, lasting some minutes, with an effort to vomit, and expectoration of viscid mucus, which can be drawn down in strings to the feet: *Kali b.*[10]

**VIOLENT** cough, in **sleep**: *Cycl.*, *Sulph.*
— — **sneezing**, cough appears in isolated attacks, is very violent, and ends with repeated sneezing: *Agar.*
— sudden cough, in morning, with stitches in side at every cough: SQUIL.
— suffocative cough: Ipec.[5]
— **sulphur** vapor, violent cough with sensation as if caused by, followed by dullness of head and headache: Brom.[6]
— cough, from dryness of throat: Kali chlo.
— —, first dry, afterward accompanied by frequent, saltish expectoration and pain as if something were torn in throat: Calc.[6]
— — from water getting into trachea: Spig.
— — **yawning**, agg.: Mur. ac.
— — after **waking**: Rhus.
— — on **waking**: Calc., Carbo v.—6.
— — entering warm room: *Natr. c.*
**VIOLIN**, playing on, agg. cough: Calc., Kali c.—5.
— — see also Music.
**VISION** cloudy, when coughing: Coff., Kali c., Lach., Sulph.—11.
— see under Eyes.
**VOICE** deep and hoarse: Iod.[5]
— inaudible from coughing: Merc.[5]
— loss of, remaining after cough: Phosp.[5]
**VOMITING** with or from coughing: ALUM.,[1] *Anac.*, ANT. T., Arg. n.,[1] Arn., Bell., BRY., Calc., Cann. s., Caps., *Carbo v.*, Coc. c., Con., Cupr., *Daph.*,[12] Digit., DROS., *Ferr.*, *Hepar*, Hyos., Indigo, Iod., *Ipec.*, Kali b., Kali c., *Lach.*, Merc., Merc. c.,[1] Mezer.,[1] Millef., Natr. c., Natr. m., Nitr. ac., *Nux v.*, Phosp.,[1] *Ph. ac.*,

**VOMITING**—(*Continued*).
Plumb.,[1] *Puls.*, *Rhus*, *Sabad.*, Sep., *Sil.*, *Sulph.*, Sul. ac.,[1] Tarent.,[1] Thuja,[1] Verat.—5.
— — continued dry hacking cough, with vomiting and arrest of breathing: ALUM.
— — continuous cough, with sweat on forehead, shocks in head, retching and vomiting: *Ipec.*
— — dry cough, with perspiration and lachrymation, stitches in vertex, vomiting and pain in stomach: Sabad.[10]
— — cough causes vomiting; no nausea: IPEC.
— — cough, with vomiting of food, nausea, and headache, or involuntary urination: Ph. ac.
— — spasmodic cough, with gagging and vomiting of ingesta and sour phlegm (3 A. M.): *Kali c.*
— — splitting headache, child hold its head, face blue, with vomiting in mornings: *Nux v.*[7]
— — violent cough inducing vomiting, or a suffocative cough before and after going to bed, also at night: Indigo.[6]
— — vomiting remaining after whooping cough: Carbo v.[7]
— **acid**, when coughing: Natr. c., Phos., Thuja.
— — **acrid**, morning, on coughing: Thuja.
— **bilious** (bitter): Carbo v.,[7] Cham., China, Cina, Cupr., Lach., Merc., Mezer., Puls., Sabad., Sepia, Stram., Sulph.—2.
— — dry cough, especially in evening in bed, till midnight, frequently with nausea and bitter vomiting: Sepia.[10]
— — followed by vomiting of ingesta: Bry.[2]
— — — — — — blood or mucus: Carbo v., Verat.—2.

**VOMITING bilious** at night: Merc.[2]
— **bloody**: Arn., Carbo v., China, Cupr., Dros., Hyos., Ipec., Nux v., Sulph.—2.
— — dark clotted blood: Nux. v.[2]
— — cough with vomiting and yellowish frothy expectoration, mixed sometimes with streaks of blood; the cough fatigues and hinders sleep: Daph.
— **eating**, some hours after eating: Meph.
— — cough after eating, with vomiting of food: *Anac.*
— — cough after eating or drinking, with vomiting of ingesta; worse on going into warm room: Bry.
— — cough and vomiting after the other symptoms of whooping cough are gone, especially after a full meal: Carbo v. (Nash.)
— — cough after a meal, with vomiting of food, especially after cold fluids; pains in arms and shoulders: Digit. (Nash.)
— — spasmodic cough, ceasing immediately after a meal, or else commencing after a meal, with vomiting of food: Ferr.
— — cough when eating or drinking anything hot; must cough until food is vomited: *Mezer.*
— **evening**: Carbo v., *Coc. c.*
— of **food**, then of blood: Nux v.[2]
— — — then of bile: Bell., Digit., Lyc., Natr. m., Phosp., Samb.
— — — then bile, next of water: Ipec.[2]
— — — then of mucus: *Dros.*, Nux v., Puls., Sil.—2.
— — — — — bitter mucus: Sil.[2]
— **inclination** (desire for) to: Ars.,[12] Bell.,[12] Brom., Bry., Caps., Cimex, Cina, Coc. c., *Dros.*, Hepar, Iod., Ipec., Kali b., Kali c.,[12]

**VOMITING**—(*Continued*).
Lach.,[12] Merc., Nux v., Petro.,[12] Ph. ac., Puls., Ruta, Senega, Sepia, Squil., Verb.—2 and 5.
— — — dry severe cough, mostly in morning with retching and desire to vomit, and sensation as if stomach were turned inside out: Puls.[10]
— of **ingesta** (in general): Anac., *Ant. t.*, Arn., Ars., Bry., Calc., Carbo v., Cina, Dig., Dros.,[12] Eugen.,[12] Ferr., Hepar, Hyos., Ign., Iod., Ipec., Lach., Laur., Meph., Mezer., Natr. m., Nitr. ac., Phos., *Ph. ac.*, Rhus, Stann., Sulph., Verat.—2 and 5.
— — some hours after eating: Meph.[2]
— — every time one drinks: Dros., Verat.—2.
— — evening: Carbo v.,[2] Rhus.[10]
— — when lying on back: Rhus.[2]
— — about midnight: Ferr.[2]
— — of tough white ropy mucus, later of food, especially in morning when trying to expectorate tough mucus: Coc. c.[10]
— — only of solid ingesta: Bry., Cupr., Verat.—2.
— — — — liquid ingesta: Acon., Ant. cr., Cham., Ipec., Nux v., Sil., Spongia.—2.
— — of cold drinks: Sil.[2]
— — — — — after they have become warm in stomach: *Phos.*
— — cough with vomiting of food and sweat on forehead: Ant. t.[10]
— **milk**: Chelid.,[1] Spong.[2]
— **midnight**, vomiting from cough before: Arg. n.,[1] Ferr.[2]
— **morning**: Kali c., Nux v.,[7] Silicea,[7] Sulph.—12.
— **mucous**: Carbo v., Cham., Cina, Coc.c.,[10] Con., Coral., Dros.,[1] Dulc., Hyos., Ipec.,[5] Mezer., Natr. c.,[1] Puls.,[5] Sil.,[12] Seneg., Spong., Stram.—2.

**VOMITING mucus** of sweetish taste: Calc.[2]

— — succeeded by vomiting of blood or bile: Verat.[2]

— — spasmodic cough with gagging, or vomiting of ingesta and sour phlegm: Kali c.[7]

— of **purulent** matter, when coughing: Sil.[6]

— **saliva**, when coughing: Cimex.[1]

— of **water**: Caust.,[2] *Dros.*,[1] Mezer.,[2] Natr. c.[1]

— — — then of ingesta: Ipec., Nux v., Sep., Sil., Sulph., Sul. ac.—2.

— — — then of solid ingesta: Sulph.[2]

— — — — — bitter tasting ingesta: Sil.[2]

— of **worms**: Cina.[2]

**VOMITURITION** (retching): Ant. t., Bell., Brom., Carbo v., China, Cupr., *Dros.*, *Hepar*,[12] Hyos., Iod., *Ipec.*, Kali c., Kreos., Merc., Mezer., Natr. m., Nux v., *Puls.*,[12] Senega, Sepia, Squil., Stann., *Sulph.*, Verat.—2.

— dry severe cough, mostly in morning, with retching and desire to vomit, and sensation as if stomach were turned inside out: Puls.[10]

— cough caused by dryness and scraping in chest, with nausea and straining to vomit: Puls.[10]

**YAWNING** excites cough: Arn., Cina, Mur. ac., Nux v., Staph.—12.

— and coughing consecutively: Ant. t.

— with (or after) cough: Anac., Ant. t., Brom., Ign., Kreos., Lobel.,[7] Nux v., Op., Phos., Rhus, Zinc.—5.

— and sudden loud cry, after cough: Op.[6]

— and screaming: Op.[8]

**WAKENED** by the cough: Apis,[1] Bell.,[7] Caust., Cocc., *Coc. c.*,[1] Coff.,[6] Daph., Dros.,[6] Graph.,[6] Hepar, *Hyos.*, Kali c., Lach., Mag. m., Nitrum, Op.,[1] PHOS.,[5] *Rhus*, *Sang.*, *Sepia*, Sil.,[1] Squil.,[7] *Sulph.*, Stront., Zing.[1]—12.

— compare with Sleep disturbed by Cough.

— at 2 A. M.: Cocc., Dros.,[7] Nitrum.—5.

— 3 A. M., cough wakes at, with stupefying headache; worse if she moves or raises herself: Nitrum.

— anxious, painful, or short cough, waking before midnight: *Rhus.*

— barking cough, waking suddenly at 11 P. M.; face fiery red; crying: Bell.[7]

— constant cough wakes patient, at night: SEPIA.

— cough after lying down, prevents sleep, and wakens him toward midnight, with some distinct clicking down back, which irresistibly irritates to violent shocks in head; ceases after loosening a small lump of mucus, which is swallowed: Apis.

— dry cough at night, waking from sleep, with intense pain in chest, when coughing; little cough during day: Kali c.[6]

— dry cough, awakening and not ceasing until he sits up in bed and passes flatus upward and downward: *Sang.*[7]

— dry cough at night, waking, must rise up: Mag. m.

— dry cough, with tickling and scraping in larynx, woke him from sleep very early in morning: *Opium.*

— dry troublesome cough, causing soreness in forepart of chest, that woke her: PHOS.

**WAKENED**, dry cough at night, waking from sleep: SULPH.

— dry cough caused by scratching in larynx, wakens patient at night: *Zing.*

— frequent cough at night, wakes him, after which he again falls asleep; almost incessant cough while lying, disappears on sitting up: HYOS.

— short, anxious, painful cough, that frequently awakens her before midnight, with very short breath: *Rhus.*

— wakes at night, from sneezing, with tickling in throat causing cough; pain in back: Amm. m.[7]

— shrill cough, waking from sleep: Sol. t. æ.

— tickling cough, wakes him: Lach.

**WAKING**, cough on: Acon., Ambra, Apis, Aralia, Bell., Calc.,[6] Caust., Chel.,[1] *China*, Cina, Coc. c., Cot., Dig., Euphr., Ign., *Kali b.*, Kreos., Lac. can., Lach., Lachn., Natr. ars.,[1] Nitr. ac., Nitrum, Nux v., Ph. ac., Psor.,[7] Puls., *Rhus*, *Sang.*, Sepia, Sol. t. æ.,[1] Spong., Squil., Stram., Sulph.,[10] Sul. ac., Tarent.[1]—5.

— **anxious** cough, on waking before midnight: *Rhus.*

— **convulsive** cough, morning, on waking: Thuja.

— — — from irritation in larynx, night, on waking: *Thuja.*

— **dry** cough, on waking: Agar., Bry., Coc. c., Dign., *Sang.*, Sol. t. æ.

— — — afternoon, on waking: Thuja.

— — — morning, on waking: Caust., Ign., Mag. s., Sil.

— — — night, on waking: SULPH.

— **exhaustive** cough, morning, on waking: Thuja.

— — — — after waking: Mag. s.

**WAKING**, **hacking** cough, on waking: Phos.

— — — agg. morning, on waking: Sil.

— **hollow** cough, morning, on waking: IGN.

— **irritation** to cough, night, on waking: Thuja.

— **midnight**, cough on waking at: Sulph.

— — cough, after having slept an hour or two awakes patient, occurring before: *Aralia.*

— **morning**, cough, on waking: Ailan., Cod., Kali b.,[7] Sulph., Tarent.

— — — agg. in: Psor.,[7] *Sil.*[1]

— — cough at 3 A.M., most severe after waking: Rhus.[13]

— — cough worse evening and before midnight, or in morning soon after waking: Rhus.[7]

— — cough with tough yellow sputa, on waking in: Aurum.[7]

— — chronic loud cough from stuffing at the epigastrium, chiefly on waking in the morning; has then a fit of coughing and expectoration of tough mucus, with lightness in the head: Kali b.[6]

— — convulsive cough, on waking: Thuja.

— — dry cough, on waking: Caust., *Ign.*, Mag. s., Sil.

— — — must sit up on account of dry, fatiguing cough, morning, after waking: Mag. s.

— — exhausting cough, on waking: Thuja.

— — — — after waking: Mag. s.

— — hacking cough, agg. on waking: Sil.

— — hollow cough, on waking: IGN.

— — paroxysmal cough, after waking: RUMEX.

**WAKING**, **morning**, racking cough, on waking: Caust.
— — tickling cough, after waking: Carbo v.
— **night**, dry cough, whenever he wakes from sleep, disappearing while sitting up in bed, and returning as soon as lying down again: *Puls.*[10]
— — on waking she is affected with cold, swelling of pit of stomach, violent cough, difficult expectoration, arrest of breathing, and sweat as from anguish: Carbo an.[6]
— **paroxysmal** cough, morning after waking: RUMEX.
— **racking** cough, morning, on waking: Caust.
— **short** cough, on waking, 7 A. M.: Dign.
— **tickling** cough wakens him, when falling asleep: *Lach.*[10]
— **tickling** cough, morning, on waking: Carbo v.
— — — with taste of blood, on waking: Ham.[7]
— **violent** cough, after: Rhus.
— — immediately on waking, violent wheezing and panting, then violent cough, causing him to sit up and bend forward: Kali b.[6]

**WALKING** excites (or agg.) cough: Alum., Ars., Carbo v., Cina, Dig., Ferr., Hepar, Iod., Lach., Natr. m., Rumex, Senega, Stram., Stront., Sul. ac.—5.
— **amel.**, dry cough on rising going off on walking: Grat.[6]
— — cough did not trouble him while walking; but as soon as he sat down it returned. Canc. f.
— **afternoon**, dry cough on walking in the: Thuja.

**WALKING**, cough torments him most while: Hepar.[6]
— cough **after**: Mezer., Natr. ars., Stront.
— in **cold air** agg. cough: Ars., Ipec., Phos.,[10] Verat.[7]—1.
— — dry tickling cough after walking in sharp, cold air: Verat.[7]
— — cough continuing without an interruption after walking in cold air, and when lying down, morning and evening, excited by deep inspiration; accompanied by colic as if umbilicus would be torn out, heat in face and sweat on forehead: Ipec.[6]
— in **open air**, cough on: Acon., Ang.,[6] Ars., Carbo v., Cina, Dig., Ferr., Ipec., Lyc., Nux v., Ox. ac.,[7] Phos., Ph. ac., Seneg., Spig., Staph., Stram., *Sulph.*, Sul. ac.
— — — dry cough after a meal, or only when walking in open air: Sulph.[10]
— — — cough from tickling in larynx when walking in open air; larynx feels swollen: Ox. ac.[7]
— — — hacking cough, agg. when: Osm.
— — — — — tickling in larynx felt only when walking in open air, causing hacking cough: Angust.
— — — — short cough: Angust.
— **fast** excites cough: Merc., Natr. m., Seneg., Sil., Squil., Stann.—2.
— — after, in open air, cough: Sepia.
— — agg. hacking cough: Senega.
— in **hot** sun, causes paroxysmal cough: Coca.
— in **morning**, amel. dry cough: Grat.[1]

**WARM air** agg. or excites cough: Ant. cr., Iod.—2.

**WARM air** agg. cough. Compare with Warm Room.
— — amel. cough: Phosph., *Rumex.*
— — cough on going from warm to cold air: Verat. v.[7]
— on **becoming**: Caust., Nux m.
— **bed**, becoming warm in, excites or agg. cough: Ant. t., Brom., CAUST., Cham., Dros.,[12] Led., Merc., Natr. mur., *Nux mos.*, Nux vom., *Puls.*, *Verat.*—5.
— — becoming, in, amel. cough: Cham.,[2] Kali b.[7]
— — cough on becoming warm in, or after recovering natural warmth from a colder state: *Caust.*[14]
— — on becoming warm in, evening, cough with profuse tough expectoration: Nux mos.[2]
— — excites paroxysmal cough: Naja.
— **exercise**, cough with or without expectoration when becoming warm in bed, or becoming warm from work: Nux m.[10]
— **fluids** (drinks) agg. cough; Ambra, Ant. t., Caps., *Coc. c.*,[1] Ign.,[7] Laur., Mez., Stann.
— — amel. cough: Ars., Alum.,[17] Eupion,[1] Lyc., Nux v., Rhus, Sil.,[1] Spong., Verat.—(E. W. B.)
— **food**, excites cough: Bar., *Kali c.*, Laur., *Mez.*, Puls.—12.
— — amel. cough: *Spong.*[7]
— **room**, agg. cough: *All. c.*,[2] Ambra, Arn., Ars., Brom.,[7] Bry., *Coc. c.*, Dig., Dros., Dulc., Ipec., Laur., Lyc., Mez., *Natr. c.*, Puls., Seneg., Spong., Verat.—5.
— — cough in; amel. in cold room: Coc. c.[10]
— — entering warm (air) room from open air, agg. cough: Acon.,[12] All. c.,[2] Ant. cr.,[7] Bov.,[1] *Coc. c.*, *Natr. c.*, Sep.,[1] Sulph.,[1] Verat., Verb.[2]—10.

**WARM room**, entering warm room from cold air, cough from tickling in chest, morning: Bov.
— — entering warm room, tickling in trachea, evening, dry cough: Com.
— — — —, afternoon, agg. dry cough: Anth. n., *Natr. c.*
— — — —, afternoon and evening, agg. violent cough: *Natr. c.*
— — on entering warm room from cold air, feels sensation in trachea as if full of smoke, which excites cough; feels as if he could not inhale sufficient air: Bry.[6]
— — going from warm room to open air, excites cough: *Acon.*
— **room**, going from, to cold air, or *vice versa*, causes coughing: All. c.,[2] Natr. c.,[10] Nux v.,[10] Sepia.
— — is unable to speak in hot room for incessant coughing: Coc. c.
— — violent cough come on in afternoon; it went off instantly as soon as she went into open air, but if she returned to warm room, cough returned with increased violence: Sulph.

**WASHING** agg. cough: Ant. cr.,[5] Ars., Calc., Rhus, Stram., Sul. ac., Verat., Zinc.—12.
— chest in cold water amel. cough and chest symptoms: Borax.
— dry cough from irritation in trachea as from dust, when washing, after dinner: Ferr. mag.

**WATER** drinking excites coughing: Natr. ph., Stram.
— — cold water, excites cough: Sul. ac., Verat.—7.
— — cold amel. cough: Caps., *Caust.*, Coc. c., *Cupr.*, Glon.,[1] *Opium*, Sulph.
— cough from standing in water: Nux m.[7]

**WATER-BRASH**, see Pyrosis.
— continued dry cough, early in morning, with discharge of water, like water-brash, from mouth: Bry.[6]
**WEAKNESS** from coughing: *Ars.*, Verat.
— see Debility, Exhaustion.
**WEARYING**, see Exhausting.
**WEATHER**, changes of, excite or agg. cough: Dulc.,[5] Lach., Nitr. ac., Phos., Sil., Verat., Verb.—12.
— damp agg.: Carbo v.,[5] Lach.[6]
— hot agg. cough: Lach.[6]
— stormy agg.: Mag. m., Phos., Sil.
**WEEP**, disposition to: Ant. t., Arn., Bar., Bell., Calc., Carbo an., Caust., Cham., Con., Digit., Hepar, Ign., Lyc., Mag. m., Mezer., Natr. m., Osm., Ph. ac., Puls., Rhus, Spong., Sul. ac.—8.
**WEEPING**: Arn., Ars., Bell., Cina, Hepar, Lyc., Samb., Sil., Sulph., Verat.—8. (v. Crying.)
— mood and screaming: Osm.[8]
— with whooping cough: Spong.[8]
**WET**, getting wet through excites cough: Calc., Dulc., Lach., Nitr. ac., Rhus, Sepia.—2.
**WHEEZING** cough: Acon., Ailan.,[7] Ambra, *Ant. t.*, Bell., Brom., Carbo v., China, Cina, Con., Cupr., Dros., Dulc., Gad.,[1] Hepar, Hyos., Ipec., *Kali b.*, Kali c.,[12] Kreos.,[6] Lyc., Phos., Prun. sp., Puls., *Samb.*, Sang.,[7] *Spong.*, Sul. ac.,[12] Verat.—5.
— — short wheezing or hard cough, excited by insupportable tickling in larynx, or at bifurcation of trachea, or by oppression at epigastrium, or by accumulation of mucus in larynx; worse on waking: Kali b.[2]
— panting, breathing, awakening, then cough, which compels him to sit up, bent forward: Kali b.[7]
**WHEEZING** and panting precede the cough: Kali b.[2]
— wheezy-whistling; cough begins with a wheeze and ends with a whistle; worse at night, in cold weather, and lying with head low: Samb. (Bell.)
**WHINING** with whooping cough: Ars., Cina.—2.
— mood: Acon.[6]
**WHISPERING** sound, cough has a: Card. b.
**WHISTLING** cough: Acon., Ars., Brom., Hepar,[2] Kreos., *Laur.*, Lyc.,[1] Prun. sp., Sang.[7]—12.
— hollow or whistling, spasmodic cough, excited by roughness, scratching and tickling in chest and throat; worse morning and evening, by music, by expectoration: Kreos.[2]
— frequently recurring, whistling spasmodic cough, in single coughs, excited by tickling in larynx and trachea, as if they were dry; worse day and especially toward evening: Lauro.[2]
**WHIZZING** cough, with sensation as if mucous membranes were too dry: Lauro.[7]
**WHOOPING** cough: Acon., *Ambra*, *Anac.*, Ant. cr., *Ant. t.*, *Arn.*, *Ars.*, Asar.,[16] *Bar.*, *Bell.*, *Bry.*, *Carbo an.*,[12] Carbo v., *Cham.*, China, *Cina*, *Coc. c.*, Con., Coral., *Cupr.*, *Dros.*, *Dulc.*, *Euphr.*, Guare.,[1] *Hepar*, *Hyos.*, Ign., Indigo,[12] Ipec., Kali c., Kali iod.,[1] Laur., *Led.*,[12] Lobel.,[16] *Lyc.*, Merc., *Meph.*, *Mezer.*, Mosch., Mur. ac., *Natr. m.*, Nitr. ac., *Nux v.*, Op., Par., Phell.,[12] Phos., Podo., *Puls.*, Rhus, Ruta, *Samb.*, Sang., *Senega*, Secale,[16] *Sep.*, *Sil.*, Spig.,[12] *Spongia.*, *Sulph.*, *Sul. ac.*, *Tabac.*, *Verat.*—5.

**WHOOPING** cough. Compare with Convulsive, Shattering, and Spasmodic Coughs.

— — **abdomen**, whooping cough coming, as it were, from deep in abdomen, with coughs which become gradually weaker and weaker as if from closure of the fauces as if by a plug; morning expectoration of tenacious, mixed with dark blood and having a flat taste: Ant. cr.[2]

— — **agg.** from 6–10 P. M.: Hyper.[7]

— — **afternoon**: Lyc., Mur. ac.—2.

— — — to midnight: Sulph.[2]

— — **amel.** by eating: Tabac.

— — **anxious**, before the attack of: Cupr.[8]

— — **breath**, children get stiff, breathing ceases, spasmodic twitchings, after awhile consciousness returns, they vomit and recover but slowly: Cupr.[7]

— — — with suffocating feeling: *Dros.*, *Ipec.*

— — — — — —, cannot exhale: Mephitis.[2]

— — — with whistling, sawing breathing between coughs: *Spong.*[7]

— — **body**, preceded by rigidity of body and paleness of face: Cina.[6]

— — **bronchi**, cough, deep, hollow, ringing, whooping, excited by tickling in lowest branches of bronchi; expectoration of yellow, tough, tenacious mucus, of bitter, saltish, sour or putrid taste: Verat.[7]

— — **chest**, **contraction** of, whooping cough, barking, croup-like, with suffocating contraction of chest, violent beating of heart, rattling, anxiety, congestion, blood-spitting; convulsions: *Stram.*[7]

**WHOOPING** cough, **chest**, **deep** from, whooping cough, violent, racking; seemingly from deep in chest, in paroxysms of long continuance; followed by expectoration of ropy mucus, adhering to pharynx: Lobel.[7]

— — — **irritation** in, paroxysms of asthmatic whooping cough, excited by irritation in chest; expectoration in morning of dark blood, or of a thin, yellowish, sometimes blood-streaked mucus, which generally tastes sour: Sul. ac.[2]

— — — **itching** in, violent spasmodic nightly cough (whooping cough), excited by itching (and tickling) in chest and throat, or from small dry spot in larynx; day expectoration, difficult, bloody, purulent, and offensive: Con.[10]

— — — **mucus** in, attacks of cough, like whooping cough, excited by copious, flat tasting, watery mucus, sometimes streaked with blood, which is in chest and throat, is difficult to dislodge, and can only be expectorated in morning: Euphr.[2]

— — — **rumbling**, after attacks of whooping cough audible rumbling, gurgling down in chest: Mur. ac.[7]

— — — **spasm** of, suffocating, hollow, deep whooping cough, excited by spasm of chest; day expectoration scanty, tenacious mucus, sweetish putrid or somewhat saltish taste: Samb.[2]

— — — **tickling** in, paroxysms of spasmodic cough, resembling whooping cough, the coughs follow each other in rapid succession, excited by tickling in chest from larynx to stomach; expec-

**WHOOPING** cough—(*Continued*). toration morning, evening, and at night of yellow, green or gray, of a milk colored white tenacious mucus: Sepia.[2]

— — **chest, tickling**, hollow, suffocating cough resembling whooping cough, excited by tickling in chest, throat, larynx, and supra-sternal fossa; in day with a scanty, tenacious, mucous expectoration of a bitter or offensive taste: Cham.[2]

— — — — spasmodic (whooping) cough, two paroxysms follow one another very rapidly, excited by tickling in larynx and upper part of chest; expectoration of acrid yellowish mucus, which is sometimes mixed with coagulated blood, tasting putrid or salty; cough is at night: Merc.[10]

— — — — whooping cough, excited by tickling in chest; in afternoon and evening without, in morning, with slight dislodgment of a yellow, watery mucus of a fatty taste, and which must be swallowed; sometimes dark blood: Mur. ac.[7]

— — — — whooping cough, spasmodic violent cough, excited by tickling in larynx and chest, with expectoration (except in evening) of acrid pus or grayish green, cold mucus, of putrid smell, or of pale, clotted, at times brown blood: Rhus.[10]

— — with **costiveness** and loss of appetite: Podo.[10]

— — **croupish**, whooping cough with croupish hoarseness of the cough: Brom.[7]

— — — whooping cough, croupish sound, pain in larynx, choking from mucus in larynx; worse morning: Hepar.[7]

**WHOOPING** cough, **croupish**, whooping cough, even of sucklings, sounding like croup; rattling breathing, anxious and restless, chest and abdomen hot, urine pale: Asaf.[7]

— — **day** agg. in: Brom., Cupr., *Euphr.*—2.

— — **dry** whistling, no sputum; impending paralysis of lungs: Lauro.[7]

— — with **fear** and terror: Spong.[8]

— — with **hæmorrhage**, from mouth, nose, with anguish, blue face, wheezing breathing and suffocation: Dros.[6]

— — — with nose-bleed, bleeding from mouth, vomiting; loses breath, turns pale or blue, and becomes rigid: Ipec.[7]

— — — bleeding from eyes, nose and mouth, face blue, splitting headache, child holds head; worse morning: Nux v.[7]

— — deep **inspiration** agg. panting cough, like whooping cough: Dulc.[7]

— — **evening**: Ambr., China, Cina, Dros., Ign., Lyc., Senega.—2.

— — — lying down: Coc. c., Dros., Natr. m.—2.

— — — till midnight: Arn., Bar., Carbo v., Hepar, Mezer., Puls., Sepia, Spong., Sul-ac., Verat. —2.

— — — and night: Ars., Bry.—2.

— — **forenoon**: Sepia.[2]

— — **larynx, burning** and tickling in larynx excite shattering cough, like whooping; morning expectoration, much tenacious mucus, albumen-like, odor somewhat offensive: Senega.[2]

— — — — clear ringing or whistling whooping cough, excited by burning sticking in larynx and trachea; generally without ex-

**WHOOPING** cough—(*Continued*).
pectoration; rarely, morning and day an expectoration of mucus and coagulated blood: Acon.[2]

— — **larynx, irritation,** spasmodic hollow, whooping cough, in short hard coughs and infrequent paroxysms, excited by feeling as if sulphur vapor were inhaled, or by creeping irritation in larynx and trachea; morning expectoration of yellow, greenish, or purulent, sometimes brownish, bloody, or less frequently, a tenacious, whitish mucous or watery expectoration. The sputa have an offensive sour or saltish taste and an unpleasant odor: Carbo v.[2]

— — — — spasmodic, violent whooping cough, excited by an irritation from larynx down into chest; morning expectoration of yellow or albumen-like tenacious mucus, tasting like an old catarrh or somewhat saltish: Mez.[2]

— — — — deep, hollow, barking, whooping cough, excited by an irritation high up in larynx, as if from plug or valve; attended in morning alone by the detachment of a scanty, tenacious, yellow or indurated mucus of a scarcely perceptibly sour taste, and which has to be swallowed again: Spong.[2]

— — — **mucus,** whooping cough, excited by excessive secretion of mucus in larynx and trachea; hence each paroxysm is attended by copious, easy expectoration of tasteless mucus, and often with florid blood: Dulc.[2]

— — — **plug,** whooping cough, deep, hollow, barking, caused by sensation as of a plug in larynx,

**WHOOPING** cough—(*Continued*).
with expectoration only in morning of small quantities of tough, yellow, hardened mucus, which has to be swallowed again: Spong.[10]

— — **larynx, spasms,** whooping in long, uninterrupted paroxysms, which last until the breath is completely exhausted, excited by mucus in trachea or by spasms in larynx; evening quite dry, in morning often with a scanty expectoration of mucus with dark blood, of a putrid taste and odor: Cupr.[2]

— — — **tickling,** attacks of deep, dull, whistling whooping cough, excited by tickling in larynx, which feels as if it were caused by down; morning expectoration of masses of mucus, which are often purulent and bloody, and generally, a sour, but sometimes a sweet taste, and, in latter case, an offensive odor: Hepar.[2]

— — — — whooping cough, nightly periodical attacks of cough from tickling in larynx, ending with expectoration of a large quantity of viscid, stringy mucus: Coc. c.[10]

— — — — violent, laborious whooping cough, excited by tickling in hard palate and larynx; expectoration by day and in evening of yellow or gray, after cold mucus, generally of a sour or sweetish, sometimes of a bitter, putrid, or metallic taste; or, finally, of clear dark red blood: Nux v.[2]

— — — — shattering cough like whooping cough, excited by burning and tickling in larynx; morning with much tenacious, mucous, albumen like expectora-

**WHOOPING** cough—(*Continued*).
tion, of a somewhat offensive odor: Senega.[2]

— — **larynx, tickling**, spasmodic whooping cough, in which two paroxysms occur in quick succession, followed by a longer interval; in the paroxysm the coughs follow each other rapidly; the cough is excited by tickling in larynx as if from down; morning and during day expectoration of dark blood, or of yellow, greenish, purulent, often cold, or milk-white watery mucus, of commonly a sourish or a putrid-flat or saltish taste, or like an offensive discharge of an old catarrh: Sulph.[2]

— — — — spasmodic (whooping) cough, two paroxysms follow one another rapidly, excited by tickling in larynx and in upper part of chest; cough at night, not during day, with expectoration of acrid yellowish mucus, which is sometimes mixed with blood, tasting putrid or salty: Merc.[10]

— — — — whooping cough, spasmodic violent cough, caused by tickling in larynx and chest, with expectoration (except in evening) of acrid pus, or grayish-green cold mucus, of putrid smell, or of pale, clotted, at times brown blood: Rhus.[10]

— — **midnight**, worse after: Acon., Amm. c.,[7] China, Dros., *Hyos.*, *Nux v.*, Samb.—2.

— — — wakes at 2 A. M.: Dros.[7]

— — — worse at 3 A. M., with gagging and vomiting, swelling between upper lids and eyebrows: *Kali c.*[7]

— — **before** midnight, agg.: Lyc., Mur. ac.—2.

— — — from evening till midnight: Arn., Bar., Carbo v., Hepar, Mezer., Puls., Sepia, Spong., Sul. ac., Verat.—2.

— — **morning**: Ant. cr., Cina, Verat.—2.

— — in **nervous**, thin little girls: Viola od.[7]

— — **night** agg. at: Ambra, Anac., Ant. t., Arn., Ars., Bar., Bry., Carbo v., *Cham.*, China, Coc. c., Coral., Cupr., Dros., Dulc., Hepar, Hyos, Meph., *Merc.*,[10] Mezer., Mur. ac., Natr. m., Nitr. ac.,[1] Puls., Samb., Sepia, Sil., Spong., Sulph., Sul. ac., Verat.—2.

— — with **sneezing**: Cina.[7]

— — **stomach, pain**, whooping cough, with crying, or pain in stomach before the attack, with expectoration of blood (pale or coagulated), congestion of blood to head, sparks before eyes, spasms in throat, involuntary stool and urine, oppressed breathing, stiffness of limbs, shaking of whole body, and general dry heat: Bell.[10]

— — — **stitches** in, whooping cough, violent straining and vomiting, stitches in pit of stomach, inability to breathe deeply; hiccough as if he would suffocate after the cough: Tabac.[7]

— — — **tickling** in, hollow, spasmodic (whooping) cough, caused in evening by sensation of sulphur vapor or dust in pit of throat; in morning, from a tickling above pit of stomach, with expectoration in evening, difficult and smelling like an old catarrh: Ignat.[2]

— — — — whooping cough, caused by tickling in throat or in pit of stomach, with expectoration (only in morning) of yellow or blood-streaked mucus, with vio-

**WHOOPING** cough—(*Continued*).
lent pain in head, as if forehead would burst, or with shocks; beating and hammering in head, involuntary micturition, stitches in region of liver: Natr. m.[10]

— — **throat**, constricted spasmodic action across throat beneath jaws; cough worse at night, with diarrhœa: Sang.[7]

— — —, **dust**, hollow, spasmodic (whooping) cough, caused by a sensation of vapor of sulphur or dust in pit of throat; in morning, from a tickling above the pit of stomach, with expectoration in evening difficult, tasting and smelling like an old catarrh: Ign.[10]

— — — **mucus**, true whooping cough, in violent, periodically recurring paroxysms, excited by a sensation as if down were in throat, and by a quantity of adherent mucus in throat; in evening expectoration of a whitish slimy, rarely somewhat bloody, almost tasteless substance, which is detached with difficulty: Cina.[2]

— — — — attacks of cough, like whooping cough, excited by copious, flat tasting, watery mucus, sometimes streaked with blood, in chest and throat, which is difficult to dislodge, and which can be expectorated only in the morning: Eupr.[7]

— — — **tickling**, whooping cough in violent periodical attacks, caused by a titillating sensation as of feather in throat, and also by much tough mucus; in evening difficult expectoration of white, occasionally of blood streaked mucus: Cina.[10]

— — — — croupy, rough, barking or whistling cough, excited by tickling in throat, and as if by vapor of sulphur: Brom.[2]

— — **throat, tickling** whooping cough, attacks, every one to three hours, with barking or dull sounding coughs, choking breathing, caused by tickling or dryness in throat, or from sensation of a soft feather in larynx; expectoration, only in morning, yellow and bitter, has to swallow it again: Dros.[10]

— — — — spasmodic whooping cough, as if from vapor of sulphur, or excited by tickling in throat and in epigastrium; morning and day expectoration of mucus, which is yellow or mixed with coagulated brownish blood, often cold; has generally an unpleasant flat taste, and is at first difficult to dislodge: Bry.[2]

— — — — whooping cough, coming from deep in chest, excited by violent tickling in throat, in rather long paroxysms; in morning, with expectoration consisting, generally, of grayish white, seldom of yellow mucus, of a salt or sour taste: Ambra.[2]

— — — — powerful spasmodic nocturnal paroxysms of whooping cough, excited by tickling and itching in chest and throat, or as if by dry spot in larynx; in day, with a difficult, bloody-purulent, sometimes hardened expectoration, of a putrid taste and smell: Con.[2]

— — — — spasmodic whooping cough, excited by tickling in throat and in epigastrium; in morning, with expectoration of a yellow mucus, often streaked with blood, generally with a flat,

**WHOOPING** cough—(*Continued*). sometimes sourish, more rarely a saltish taste: Natr. m.[2]

— — **throat, tickling** spasmodic cough, like whooping cough, excited by tickling and roughness and in throat and in epigastrium; morning expectoration of difficult, yellow, tenacious, starch-like, often saltish mucus: Baryt.[2]

— — — — violent, laborious whooping cough, excited by tickling in hard palate and larynx; by day and in evening with expectoration of a yellow or gray, often cold mucus, generally of a sour or sweetish, sometimes of a bitter, putrid, or metallic taste, or finally, of a clear, dark red blood: Nux v.[2]

— **trachea, itching** in, shattering, spasmodic whooping cough, often in paroxysms of two coughs each, excited by an itching scratching, with a feeling of dryness, and, as it were, of sulphur vapor in trachea and chest; in morning and day, with expectoration of much yellow or greenish mucus of various tastes: Puls.[2]

— — — **irritation**, whooping cough excited by irritation in trachea, as if from sulphur vapor; in morning, and during day, with a purulent (lemon-yellow, gray, greenish or whitish), or bloody mucous expectoration, of a salt (also sometimes a bitter, flat, putrid, sweetish or sour) taste and offensive odor: Lyc.[2].

— — — — spasmodic, hollow, whooping cough, in short hard coughs and infrequent paroxysms, excited by a feeling as if sulphur vapor were inhaled, or by a creeping irritation in larynx and trachea; in morning, with a

**WHOOPING** cough—(*Continued*). yellow, greenish, or purulent, sometimes brownish bloody, expectoration, or, less frequently, a tenacious, whitish mucous or watery expectoration; sputa has an offensive sour or saltish taste and unpleasant odor: Carbo v.[2]

— — — **mucus**, whooping cough in long, uninterrupted paroxysms, which last until the breath is completely exhausted, excited by mucus in trachea or by spasms in larynx; in morning, of a scanty expectoration of mucus with dark blood, of putrid taste and odor: Cupr.[2]

— — — — whooping cough excited by excessive secretion of mucus in larynx and trachea; hence each paroxysm is attended by copious, easy expectoration of tasteless mucus, and often with florid blood: Dulc.[2]

— — — burning **sticking**, a clear, ringing, or whistling whooping cough, excited by burning sticking in larynx and trachea; generally without expectoration; or, rarely, in morning, and during day, with expectoration of some mucus mixed with coagulated blood: Acon.[2]

— — — **tickling**, whooping cough which shakes the patient thoroughly; paroxysms every three to four hours, excited by tickling in trachea (after the attack yawning and sleepiness[10]); during day, with expectoration of mucus, which generally has a sweetish flat taste, is often bloody; at other times, yellowish purulent and sometimes gray and acrid: Anac.[2]

— — — — clear-ringing, crowing or whistling whooping cough, ex-

**WHOOPING** cough—(*Continued*). cited by a burning tickling in trachea and in throat-pit, as if from vapor of sulphur; in day with expectoration of mucus, scanty and generally frothy, or in lumps of various taste and color; returning periodically, with increasing violence: Ars.[2]

— — **trachea, tickling,** a hollow suffocating cough, resembling whooping cough, excited by tickling in chest, trachea, larynx and supra-sternal fossa; in day, with a scanty, tenacious, mucous expectoration of a bitter or offensive taste: Cham.[2]

— — — — hoarse whooping cough, excited by tickling in trachea, or as if from vapor of sulphur; in day and evening, with an expectoration of pus, mixed with dark coagulated blood, or of tenacious mucus, having a flat, saltish or sour taste, or, more rarely, a repulsive sweetish taste: China.[2]

— — — — shattering, spasmodic whooping cough, with frequent, rapidly succeeding coughs, excited by tickling as if from mucus firmly seated in trachea; in day, with expectoration of a somewhat saltish mucus, or of a bright red blood mixed with coagula: Hyos.[2]

— — — — spasmodic whooping cough, severe, dry, racking, excited by tickling in right side of trachea below larynx; splitting frontal headache: Sticta.[7]

**WIND,** belching of, after coughing: Sul-ac.

— — see Eructation.

— in the, cough: Cham., Euphr., Lyc, Stram.—2.

— **cold,** cough from: Lyc.

— — cool evening, paroxysmal cough from: Coca.

— **dry** cold: Acon., Cham., Hepar, Spong.—5.

— **east**: Acon., Cham., Cupr., Hepar, Samb., Spong.—5.

— **north**: Acon., Caps.,[7] Cham., Cupr., Hepar, Sepia, Spong.—5.

— **sea** wind: Cupr.[7]

— in **sharp**: Caps.[7]

— **walking** in wind: Stram.[7]

**WINE** agg. or excites cough: Acon., Arn., Borax, Ferr., Ign., Led., *Stann.*, Stram., Zinc.—12.

— **amel.**, in afternoon severe cough, with little expectoration; on coming into warm room and smoking tobacco, cough becomes worse; when in open air and drinking wine in evening, it is diminished: Sulph.

— sour, agg. cough: Ant. cr.[2]

**WINTER** cough: Acon., Cham. —12.

**WORM** symptoms, with cough: Cina,[2] Mag. c.,[5] Spig.[7]

**WRITING** agg. cough: Cina.

